# One-dimensional fluorescent covalent organic frameworks rich in exposed sp^3^-N sites for ultra-fast iodine capture and visual monitoring

**DOI:** 10.1039/d5sc05993c

**Published:** 2025-09-29

**Authors:** Ke Li, Bing Yan

**Affiliations:** a Shanghai Key Lab of Chemical Assessment and Sustainability, School of Chemical Science and Engineering, Tongji University Siping Road 1239 Shanghai 200092 China byan@tongji.edu.cn

## Abstract

It is important to capture radioactive iodine, especially highly volatile CH_3_I and I_2_, from nuclear waste for pollution control. Research has indicated the great potential that covalent organic frameworks possess for iodine capture, and several covalent organic frameworks (COFs) with high adsorption capacities have been reported. However, increasing the capture rate of gaseous iodine remains a major challenge at present. To this end, researchers have improved iodine capture rates by introducing macropores, nitrogen sites, and heteroatoms. In the field of catalysis, 1D COFs have been used due to their abundant exposed sites. Inspired by this, we postulated that it is feasible to increase the exposure of N sites to increase the iodine capture rate. Herein, we report a strategy based on 1D COFs (COF-1D6 and COF-1D7) with the introduction of exposed sites, which significantly enhances the adsorption rates of I_2_ and CH_3_I, while maintaining a high loading capacity. In particular, COF-1D6 with completely exposed sites exhibited a *K*_80%_ of 1.07 g g^−1^ h^−1^, which is far higher than that of other reported COF adsorbents. The excellent fluorescence of COF-1D6 also enabled visual monitoring of the CH_3_I adsorption process. This work provides new insights into rapid iodine capture from the perspective of exposure sites.

## Abstract 2

COF-1D6 with completely exposed sites exhibited an ultrahigh *K*_80%_ of 1.07 g g^−1^ h^−1^ and acheived visual monitoring of the CH_3_I adsorption process. This work provides new insights into rapid iodine capture from the perspective of exposure sites.

## Graphical abstract

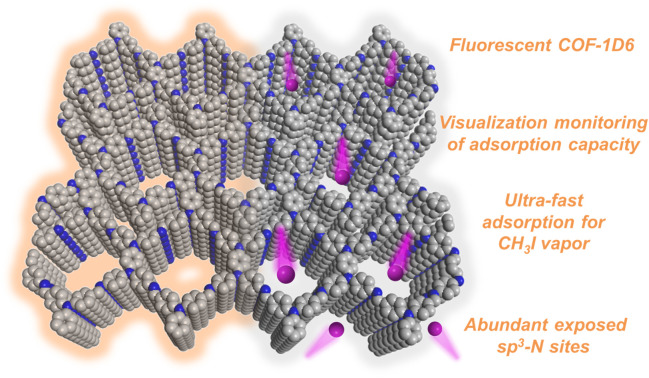

## Introduction

As the energy crisis looms, the development of new energy sources has become an inevitable necessity.^1^ Nuclear, wind, and solar energy, and other clean energy sources have become the main contributors to low-carbon power generation.^2,3^ Of these, nuclear energy has a higher energy density and controllability, and therefore, it is the best candidate to replace traditional power generation.^4^ However, radioactive pollutants (^90^Sr, ^137^Cs, ^99^Tc, ^85^Kr, and ^133^Xe) are released into the environment through accidents or improper nuclear waste processing, disposal, or storage^5–7^ Among them, ^129^I and ^131^I gravely threaten human health due to their high radioactivity, biocompatibility, and long half-life.^8–10^ Iodine commonly exists in the highly volatile form of I_2_ and CH_3_I, which may easily enter the human body through respiration and are difficult to capture.^11^ Thus, the management of radioactive iodine and the efficient treatment of gaseous iodine from radioactive waste are of paramount importance.

The traditional solution-based washing method is a cumbersome operation with high consumption costs, and therefore, using the adsorbent method is more attractive because of its high efficiency.^12^ To address the challenge of iodine capture, diverse adsorbent materials have been designed by researchers, such as silver-based nanomaterials,^13–15^ modified biochar,^16^ metal oxo clusters,^17^ zeolites,^18^ aerogels,^19^ metal–organic frameworks (MOFs),^20,21^ hydrogen-bonded organic frameworks (HOFs),^22,23^ and covalent organic frameworks (COFs).^24–27^ The adsorption of iodine mainly depends on metals that are compatible with iodine or rich N sites. However, considering the cost of precious metals and the risk of potential secondary pollution from metal ions, the design of materials rich in N-sites and non-metallic with a high adsorption performance has initiated a new era of iodine capture.^28^

COFs are outstanding representatives of crystalline porous materials^29,30^ that are widely used as adsorbents due to their stability, high specific surface area, and rich pore structure.^31,32^ The pre-design and numerous synthetic units of COFs provide a sufficient approach for the synthesis of diverse adsorbents for specific targets, by means of flexible functional group combination, and modification of different topologies and dimensions. Among the many types of COFs, imine-based COFs are synthesized through the Schiff base reaction and are rich in imine N active sites for I_2_ and CH_3_I capture.^33^ On this basis, different types of N sites can be further introduced through the flexible combination of synthetic units to enhance iodine capture capacity.^34^

Maximum adsorption capacity and adsorption rate are important indicators for evaluating iodine capture capacity. Recently, researchers have focused on enhancing the adsorption capacity by increasing the N content,^35^ and enlarging the specific surface area and pore volume, as well as introducing electron-rich impurity elements (O, S).^36^ As for adsorption rate, Lu's group increased the mass transfer rate of COF-300 for I_2_ vapor by introducing macropores.^37^ Luo *et al.* proved that the introduction of elemental S increased the efficiency of the electron formation of I_2_ into I_3_^−^ and I_5_^−^, thereby accelerating the adsorption process.^36^ Wang's research group also proposed that the N-methylation reaction rate of sp^3^-N is faster.^38^

Inspired by the above, we intended to determine the capacity and rate of COFs for iodine adsorption by the abundance of N sites, volume, and mass transfer efficiency. However, although there are abundant N sites and objective specific surface areas in common 2D COFs, their regular pore structures and strong stacking restrict the passing of I_2_/CH_3_I molecules through the pores, and then they cannot come into contact with N sites. When the pore space is narrow or blocked by iodine molecules, the adsorption efficiency is greatly reduced.^39^ Fortunately, 1D COFs formed by C_4_ units and C_2_V are widely used in catalysis,^40–42^ adsorption,^43^ and fluorescent sensing^44^ due to their abundant exposed sites, unidirectional charge transfer, and higher dispersibility.^45^

The typical band topological structure and ordered one-dimensional channels of 1D COFs significantly increase the exposure of edge sites, thereby improving the mass transfer efficiency between target molecules and N sites. Therefore, increasing the exposure of active nitrogen sites to increase iodine adsorption efficiency is feasible and has not been previously reported. Furthermore, in the actual use of adsorbents, it is very meaningful to be able to easily observe the adsorption process. Compared to 2D COFs, 1D COFs are more likely to exhibit excellent fluorescence properties,^46^ and these characteristics can be harnessed to monitor the degree of adsorption using fluorescence visualization.

Herein, inspired by the above theories and reports, we aimed to construct an efficient adsorption platform for I_2_ and CH_3_I through the strategy of exposing active sites, for achieving the following specific purposes: (i) construction of fluorescent N-rich 1D COFs, (ii) comparison of adsorption rates for I_2_/CH_3_I with different degrees of synthetic nitrogen site exposure, (iii) enhancing the adsorption rate of CH_3_I by introduction of a large number of sp^3^-N sites, and (iv) constructing a 1D COF fluorescence platform for visual monitoring of adsorption processes.

## Results and discussion

To start with, we selected C_4_ and C_2_v synthetic units for the preparation of 1D covalent organic frameworks based on previous reports and theoretical calculations. Common C_4_ monomers include 4,4′,4′′,4′′′-(pyrene-1,3,6,8-tetrayl)tetraaniline, 4′,5′-bis(4-aminophenyl)-[1,1′:2′,1′′-terphenyl]-4,4′′-diamine, tetrakis(4-aminophenyl)ethene, and *N*,*N*,*N*′,*N*′-tetrakis(4-aminophenyl)-1,4-benzenediamine and their analogues. Notably, abundant nitrogen sites are necessary for a COF to adsorb I_2_ and CH_3_I, and it has been reported that the ability of sp^3^-N to quaternize with CH_3_I is higher as compared with sp^2^-N.

**Scheme 1:**
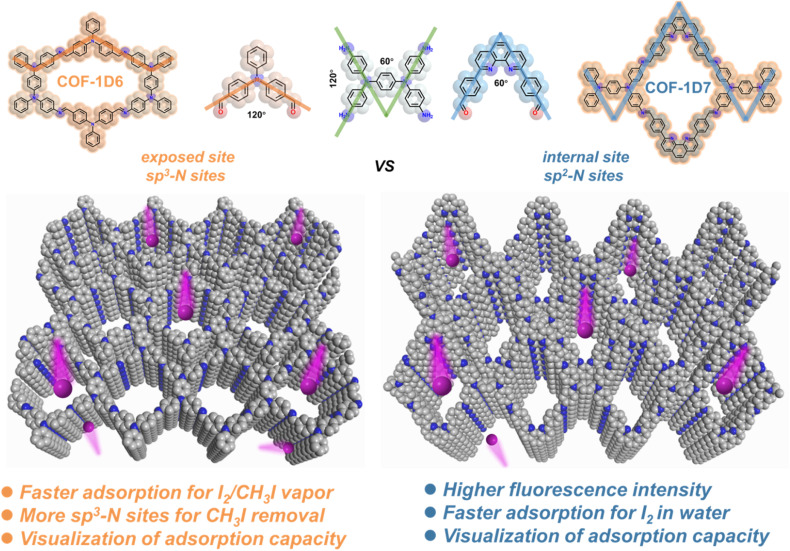
Synthetic strategies and structural skeleton of COF-1D6 and COF-1D7, and schematic diagram of the transport mode for I_2_/CH_3_I (purple balls).

To this end, we chose *N*,*N*,*N*′,*N*′-tetrakis(4-aminophenyl)-1,4-benzenediamine (TABA) because it is rich in sp^3^-N. Subsequently, 4,4′-diformyltriphenylamine (DTPA) and 2,9-bis[p-(formyl)phenyl]-1,10-phenanthroline (BPPT) were selected to prepare COF-1D6 and COF-1D7, respectively, for the following purposes. The phenanthroline group contains sp^2^-N sites and greater conjugate flatness, endowing COF-1D7 with strong π–π stacking ability that results in a more distinct pore structure. In contrast, 4,4′-diformyltriphenylamine containing sp^3^-N is not a regular plane, which may weaken the interlayer stacking of COF-1D6. Furthermore, as shown in Scheme 1, the N site in 4,4′-diformyltriphenylamine is exposed outside the COF pores, which greatly increases the possibility of contact with I_2_ and CH_3_I, and may enable COF-1D6 to achieve a higher mass transfer efficiency when adsorbing gaseous I_2_ and CH_3_I.

Thus, COF-1D6 and COF-1D7 were designed to explore the effect of N-site exposure degree on the iodine adsorption process, as well as the ability of the sp^2^-N and sp^3^-N active sites to adsorb iodine. Last but not least, DTPA and BPPT are excellent fluorescent units, and therefore, it is possible to build luminescent 1D-COFs for visual fluorescence monitoring of adsorption processes.

When constructing 1D COFs, the sum of the interior angles formed by the C_4_ unit and the V-shaped C_2_ unit should be 180°. DTPA and BPPT present different molecular angles (120° and 60°), which results in connection of the two at 60° and 120° in the direction of TABA, respectively. Fortunately, these two different connection methods allow the relatively small-size A unit to form a hole wall with the long side of TABA, while a small-size A unit forms the pore wall with the short side. With the clever combination of the three, there are similar pore sizes for 1D6 and 1D7, and this eliminates the variable of pore size for subsequent comparative experiments.

**Fig. 1:**
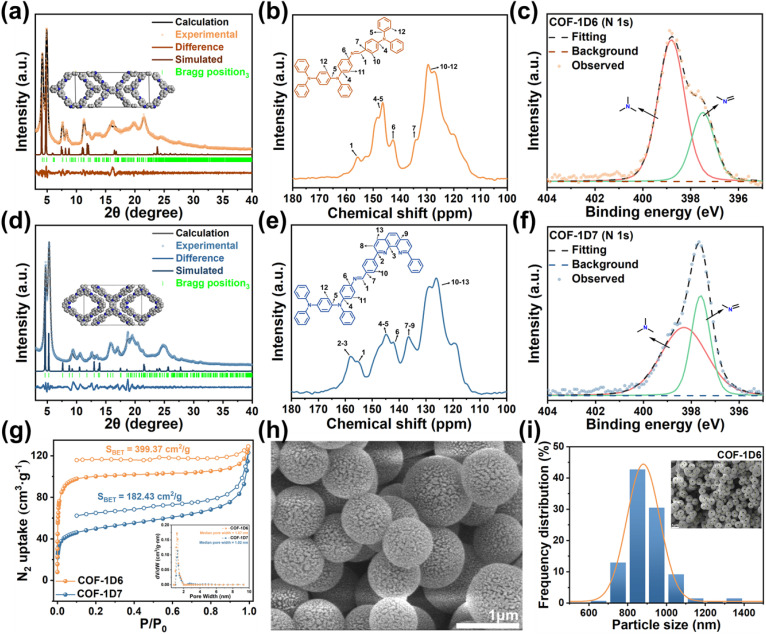
Powder X-ray diffraction (PXRD) patterns for (a) COF-1D6 and (d) COF-1D7. Solid-state ^13^C CP-MAS of (b) COF-1D6 and (e) COF-1D7. N 1s XPS spectra of (c) COF-1D6 and (f) COF-1D7. (g) N_2_ adsorption–desorption curves and pore size distribution curves (inset) of COF-1D6 and COF-1D7. (h) SEM images of COF-1D6. (i) Diameter statistics of COF-1D6.

Powder X-ray diffraction (PXRD) was carried out to investigate the crystal structure of the COFs (Fig. 1a and d). The COF-1D6 signal peak at 4.18 and 4.92° confirmed the existence of microporous structures, corresponding to the (2 0 0) and (1 −1 0) crystal plane. The remaining minor peaks at 7.40, 8.26, 8.76, 11.32, 12.00, 16.17, and 21.17° were attributed to the (3 1 0), (4 0 0), (0 2 0), (5 −1 0), (4 2 0), (7 −2 0), and (4 1 −1) crystal planes, respectively. The characteristic peaks at 4.72, 5.32, 9.42, 12.65, 13.44, 15.80, 19.52, and 24.70 for COF-1D7 correspond to the (1 1 0), (2 0 0), (0 2 0), (4 2 0), (3 3 0), (2 2 −1), (4 2 0), and (6 0 −1) crystal planes, respectively.

Then, the Materials Studio program was used to perform the geometrical optimization of the structural models, and fit experimental PXRD patterns by Pawley refinement. Unit cell parameters were obtained for COF-1D6 (*a* = 43.79, *b* = 20.30, *c* = 3.84 Å; *α* = 93.61, *β* = 98.18, *γ* = 90.48°, with a *R*_wp_ of 4.82% and a *R*_p_ of 3.67%) and COF-1D7 (*a* = 36.52, *b* = 25.15, *c* = 4.81 Å; *α* = 90, *β* = 88.10, *γ* = 90°, with a *R*_wp_ of 6.25% and a *R*_p_ of 4.85%). Furthermore, to investigate the chemical stability of the two COFs, PXRD was performed for the COFs after immersion for 48 h in water, *N*,*N*-dimethylformamide (DMF), acetonitrile (ACN), dimethylsulfoxide (DMSO), 1 M HCl, and 1 M NaOH. As shown in Fig. S3 and S4, apart from the NaOH aqueous solution and DMSO having a slight effect on the crystallinity of both COFs, the materials remained stable in the other solvents.

To explore the specific surface areas and pore sizes, N_2_ adsorption–desorption experiments were performed at 77 K to characterize the porous structures of the two COFs. As shown in Fig. 1g, the curves indicated a typical isotherm of type IV, revealing their mesoporous characteristics. Furthermore, the pore size distributions of COF-1D6 and COF-1D7 indicated that the pore sizes were approximately 1 nm (Fig. 1g inset and S5), and their median pore widths were 1.07, and 1.02 nm, respectively, proving the similarity of the pore sizes for the two COFs. In addition, the single pore size distribution indicates that both COFs are one-dimensional in configuration and extend unidirectionally. Correspondingly, the results from the Brunauer–Emmett–Teller (BET) surface area calculations were 399.27 (COF-1D6) and 182.43 (COF-1D7) m^2^ g^−1^, with pore volumes of 0.196 and 0.180 cm^3^ g^−1^ for COF-1D6 and COF-1D7, respectively.

**Fig. 2:**
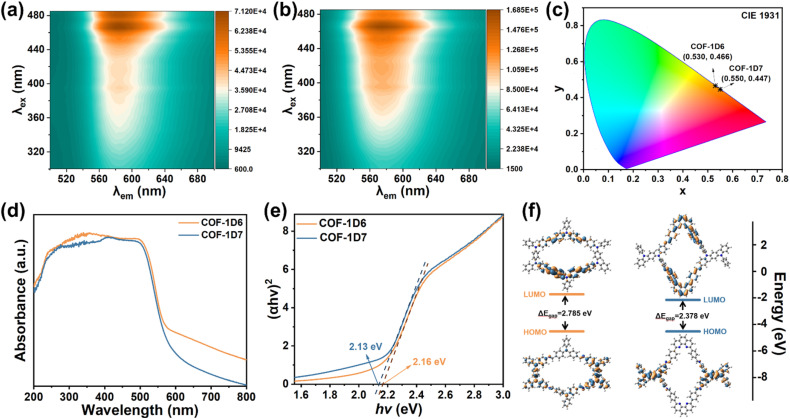
3D EEM maps of (a) COF-1D6 and (b) COF-1D7. (c) CIE chromaticity coordinates, (d) UV-Vis diffuse reflectance spectra, (e) Tauc plots, and (f) HOMO and LUMO energy levels of COF-1D6 (orange) and COF-1D7 (blue).

To further demonstrate the successful synthesis of COFs, Fourier transform infrared (FT-IR) spectroscopy was conducted on COF-1D6, COF-1D7, and their synthetic monomers (Fig. S6 and S7). In comparison to the ligands, the signal peaks at 3456–3349 cm^−1^ (–NH_2_) for TABA, 1670 cm^−1^ (–CHO) for DTPA, and 1691 cm^−1^ (–CHO) for BPPT almost completely disappeared in the FT-IR spectra of COF-1D6 and COF-1D7, which explained the thoroughness of the imine condensation reaction. Solid-state ^13^C nuclear magnetic resonance (^13^C NMR) also provided evidence for the formation of imine bonds. As shown in Fig. 2b and e, the peaks at approximately 156 ppm (label 1) corresponded to C of the imine bond, and proved that imine bonds exist in the two COFs. Furthermore, the C in COF-1D6 and COF-1D7 are in similar chemical environments, *i.e.*, the C in the C–N bond (label 4–6) and benzene ring (label 7–13).

Notably, the spectrum of COF-1D7 contains distinct peaks at 158 ppm compared with COF-1D6, and these were attributed to the C connected to N in the phenanthroline group in COF-1D7. Moreover, the X-ray photoelectron spectroscopy (XPS) also confirmed the presence of N elements in different environments (Fig. 1c and f). For this purpose, the peak of N 1s was deconvoluted into peaks of N in C=N (approximately 397.5 eV) and C–N (approximately 399.0 eV), corresponding to sp^3^-N and sp^2^-N in COF, respectively. Notably, the relative strengths of the two are reversed in COF-1D6 and COF-1D7, which is due to the fact that COF-1D6 contains more sp^3^-N units (TABA), while COF-1D7 contains more sp^2^-N units (BPPT).

Thermogravimetric analysis (TGA) was subsequently carried out to verify the thermal stability of the COFs. In Fig. S8, no significant weight loss was observed at temperatures below 450 °C, which proved that COFs have the potential for iodine capture in 80 °C iodine vapor.

Finally, we used scanning electron microscopy (SEM) to observe the morphology of the two COFs. Fig. 1h and S9 clearly show that COF-1D6 exhibits uniform spherical nanoparticles, while the morphology of COF-1D7 is not regular. Zeta potentials also showed greater particle stability for COF-1D6 (Fig. S10). This may be due to the tetrahedral configuration of sp^3^-N, which provides greater morphological plasticity to COF-1D6, allowing it to stack into a more regular spherical shape during the solvothermal reaction.

Based on the statistics of the SEM image, the diameter of spherical COF-1D6 ranges from 700 to 1000 nm (Fig. 1i). This uniform spherical morphology also facilitates comparison of properties before and after adsorption of I_2_ and CH_3_I vapor. More specific elemental content and elemental distribution were also characterized by energy-dispersive X-ray spectroscopy (EDS) and EDS mapping (Fig. S11–S13), which showed that the material elements are evenly distributed with a reasonable element ratio (C: 87.3 wt%, N: 12.7 wt% for COF-1D6; C: 89.56 wt%, N: 10.44 wt% for COF-1D7).

Considering the potential fluorescent groups in both COFs and the relatively weak interlayer stacking of 1D COFs, their PL performance was further verified by emission-excitation spectra. As shown by the 3D EEM maps in Fig. 2a and b, the optimal excitation wavelengths for both are concentrated at approximately 466 nm, while the optimal emission wavelengths are approximately 580 nm and 595 nm, respectively. Correspondingly, the CIE coordinates (Fig. 2c) for COF-1D7 are in a more red position (0.550, 0.447) compared to COF-1D6 (0.530, 0.466). Because the two COFs lack the ability to form intramolecular hydrogen bonds, the possibility of fluorescence generated by excited-state intramolecular proton transfer (ESIPT)^47^ was ruled out. Therefore, the fluorescence of the two COFs is mainly based on the intramolecular charge transfer (ICT) process, which requires the presence of a donor–acceptor pair and a fluorescent unit within the molecule.

Consistent with the pre-design of the two COFs, the combination of TABA (D), DTPA (A), and BPPT (A) satisfies the requirements for occurrence of the ICT process. Notably, COF-1D7 exhibited a higher fluorescence intensity. Solid UV-Vis diffuse reflectance spectra were used to explain the underlying mechanism behind the above phenomenon. As shown in Fig. 2d, the two COFs exhibit similar UV absorption spectra in the 200–800 nm range. After processing the spectrum by the Tauc plot method (Fig. 2e), we determined the values for the optical gap (Tauc gap) of COF-1D6 (2.16 eV) and COF-1D7 (2.13 eV).

The narrower optical bandgap indicated that COF-1D7 requires lower excitation energy, resulting in larger fluorescence emission wavelengths. In COF-1D7, BPPT is an electron-withdrawing group acting as an acceptor (A), while TABA is an electron-rich group acting as a donor (D). Under the excitation of UV light, the electrons in COF-1D7 transfer more easily from D to A, compared to COF-1D6, which contains a weak electron-withdrawing acceptor (DTPA). Because of this greater degree of electron transfer, it is easier for electrons to pass through the fluorescent group, thereby increasing the intensity of fluorescence emission.

As verification, density functional theory (DFT) calculations were performed to calculate the highest occupied molecular orbital-lowest unoccupied molecular orbital (HOMO–LUMO) energy levels for structural units of COFs (Fig. 2f). The relative magnitude of the Δ*E*_gap_ between the two (2.785 eV for COF-1D6, 2.378 eV for COF-1D7) is consistent with the Tauc gap. In the excited state (LUMO), the electrons in COF-1D7 are more concentrated in the A unit, which indicates that the electrons are more completely transferred.

The combination of theory and practice clearly illustrates that the stronger electron-withdrawing ability of the A unit leads to a narrower band gap, thereby facilitating the transfer of electrons to the A unit under excitation. When the fluorescent group is close to A or acts as the A unit itself (BPPT), the fluorescence emission intensity increases (I_1D7_ > I_1D6_). Fitting of the time-resolved photoluminescence (TR-PL) spectra at room temperature provided fluorescence lifetimes of 5.67 and 5.46 ns (Fig. S14), which corresponded to the decay of COF-1D6 and COF-1D7, respectively. The long fluorescence lifetimes indicate that both COFs have an excellent ability to provide excited electrons for the reduction process during adsorption.

**Fig. 3:**
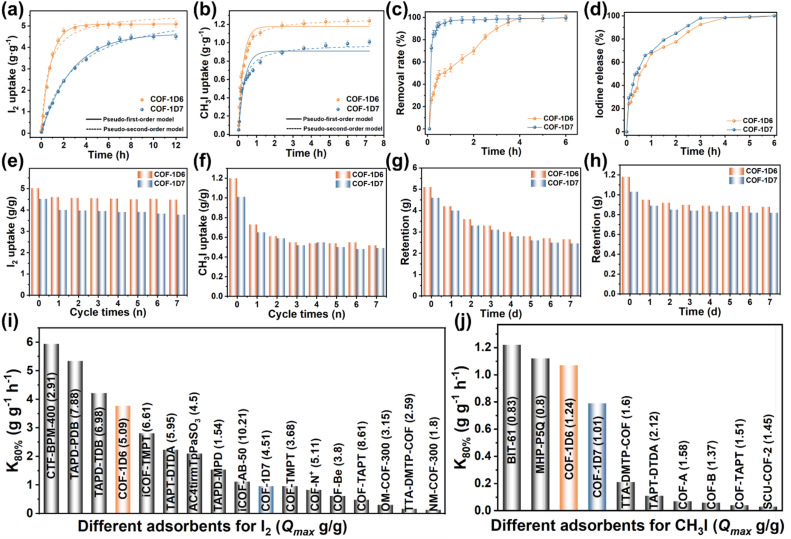
Equilibrium curves of (a) iodine vapor, and (b) methyl iodide vapor adsorption of COF-1D6 (orange) and COF-1D7 (blue) with adsorption kinetics curves fitting with the pseudo-first-order model and pseudo-second-order model. (c) Kinetic study of iodine adsorption in water (*C*_0_ = 100 ppm) and (d) iodine desorption in ethanol of COF-1D6 (orange) and COF-1D7 (blue). Regeneration cycles of COF-1D6 and COF-1D7 for (e) I_2_ and (f) CH_3_I. Retention curve at room temperature of COF-1D6 and COF-1D7 for (g) I_2_ and (h) CH_3_I. Comparison of *K*_80%_ values of several adsorbents for (i) I_2_ and (j) CH_3_I, and their adsorption capacity in brackets (corresponding references are presented in Tables S3 and S4).

Based on the structural design of the COFs and successful preparation proven by the above characterization, adsorption experiments were performed to explore the potential of COF-1D6 and COF-1D7 for I_2_ and CH_3_I capture. As shown in Fig. S15 and S16, two orange-red COFs turned black after being exposed to iodine vapor (75 °C) for a short time, demonstrating extremely strong iodine capture capabilities. COF-1D6 and COF-1D7 reached adsorption saturation at approximately 2 hours and 7 hours (Fig. 3a), with adsorption capacities of 5.09 and 4.51 g g^−1^ and a *K*_80%_ (evaluated at 80% of the full capacity) of 3.77 and 0.962 g g^−1^ h^−1^, respectively.

Fast adsorption rates and high adsorption capacities enable COF-1D6 to perform in a manner comparable to common high-performance iodine adsorption materials (specific adsorption rates and adsorption capacities are shown in Fig. 3i and Table S3). Adsorption kinetics fitting shows that the adsorption equilibrium curves of the two COFs are more consistent with a pseudo-first-order kinetic adsorption model (*R*^2^_COF-1D6_ = 0.998, *R*^2^_COF-1D7_ = 0.995), and explains that iodine vapor is adsorbed by COFs in the form of physical enrichment. Because iodine also causes significant visual pollution in water, we investigated the efficiency of the two materials for iodine removal in water (Fig. 3c and S17). Contrary to I_2_ vapor capture, COF-1D7 exhibited a higher removal rate, which stems from the greater dispersibility of COF-1D7 in water as compared to COF-1D6.

The large number of sp^3^-N sites introduced in our structural design enabled efficient adsorption of CH_3_I. Because methyl iodide is more volatile than iodine vapor, it is imperative to investigate the adsorption capacity of the two COFs for methyl iodide at room temperature. It is worth mentioning that both COFs showed extremely short equilibrium times, especially for COF-1D7, reaching adsorption saturation within two hours (Fig. 3b). Unlike I_2_ adsorption, the adsorption kinetics curve of CH_3_I is more consistent with a pseudo-second-order kinetic adsorption model (*R*^2^_COF-1D6_ = 0.994, *R*^2^_COF-1D7_ = 0.957), indicating that the adsorption of CH_3_I is mainly a chemical process. The maximum adsorption of COF-1D6 and COF-1D7 reached 1.24 and 1.01 g g^−1^, exhibiting an ultra-high *K*_80%_ of 1.07 and 0.79 g g^−1^ h^−1^, respectively. Because of such high adsorption rates and substantial adsorption capacities, COF-1D6 and COF-1D7 are the fastest COF materials for CH_3_I adsorption, second only to metal–organic framework adsorbents (BIT-61)^48^ and multi-microporous organic polymer adsorbents (MHP-P5Q)^49^ (Fig. 3j and Table S4).

The recyclability of adsorbents is an important indicator of their practical application capabilities. We first conducted iodine desorption experiments in ethanol and monitored the desorption process through UV-Vis spectroscopy (Fig. S18). As shown in Fig. 3d, both COFs loaded with iodine can reach desorption saturation within three hours. Based on their desorption performance in ethanol, the two COF materials were used in seven cycles of I_2_ adsorption and desorption (washed with *n*-hexane). Both COFs maintained 85% of their maximum adsorption capacity in the seventh cycle (Fig. 3e). However, CH_3_I retained only approximately 50% of its maximum after the second cycle (Fig. 3f), which may be due to chemical interaction with parts of N sites on the COFs. After maintaining the two materials loaded with I_2_ and CH_3_I for seven days, approximately 40% of the I_2_ escaped from both COFs (Fig. 3g), while less than 20% of the CH_3_I escaped (Fig. 3h). This shows that the retention capabilities for I_2_ and CH_3_I at room temperature for both COFs are satisfactory.

Both materials exhibited excellent adsorption properties. COF-1D6 demonstrated a higher adsorption capacity and faster adsorption rates for I_2_ and CH_3_I vapors, while COF-1D7 exhibited faster removal rates for I_2_ in water, offering significant potential for practical applications in iodine capture. Once the adsorbent reaches adsorption saturation in actual application, it must be replaced to achieve the goal of continuous treatment of pollutants. Therefore, it is essential to find a convenient and intuitive method to monitor the adsorption process.

**Fig. 4:**
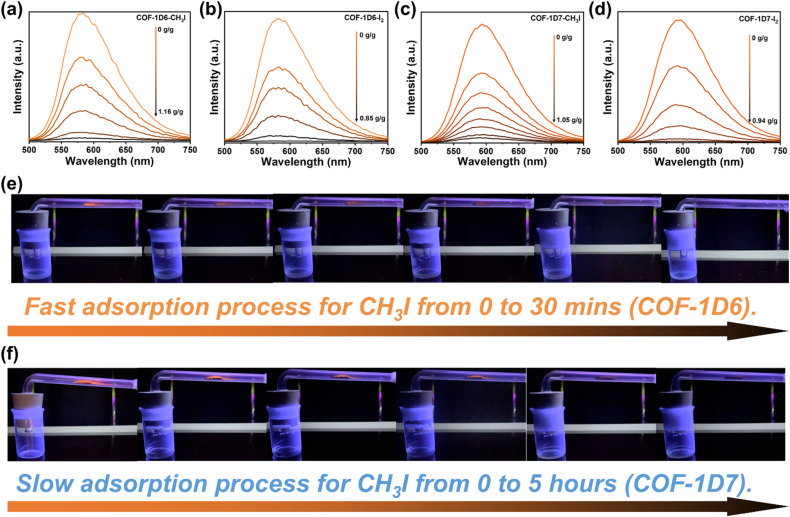
Trend of fluorescence intensity with changes in adsorption capacity of COF-1D6 for (a) CH_3_I and (b) I_2_ and COF-1D7 for (c) CH_3_I and (d) I_2_. Visual monitoring of the CH_3_I adsorption process of (e) COF-1D6 and (f) COF-1D7.

Fortunately, because both adsorbents exhibited excellent fluorescence properties, it was possible to determine the degree of adsorption based on fluorescence changes. As shown in Fig. 4a–d, the fluorescence of COF-1D6 was completely quenched after adsorption of 116 wt% CH_3_I and 85 wt% I_2_. Similarly, the fluorescence of COF-1D7 also thoroughly disappeared after adsorption of 105 wt% CH_3_I and 94 wt% I_2_. For I_2_, when the fluorescence of the adsorbent was completely quenched, only approximately 20% of the maximum adsorption capacity was reached, and therefore, it is not possible to effectively monitor the entire adsorption process. Conversely, for the adsorption of CH_3_I, fluorescence quenching precisely corresponded to the saturation adsorption amount. Therefore, it is entirely feasible to monitor CH_3_I throughout the process using the fluorescence quenching of COF-1D6 and COF-1D7.

To this end, we constructed a simple glass tube sealing device for direct capture of real-time fluorescent images under UV light (360 nm) (Fig. 4e and f). The fluorescence emitted by COF-1D6 completely disappeared within 30 minutes, as shown in Fig. 4e, indicating that COF-1D6 reached adsorption saturation in this device in only 30 minutes. As a control experiment (Fig. 4f), COF-1D7 required 5 hours to be completely extinguished under the same conditions. The adsorption rate observed by fluorescence quenching and the adsorption equilibrium curve obtained by the weighing method showed similar trends. The above clearly demonstrates that the fluorescence of COF-1D6 can be used for efficient, visual monitoring of adsorption processes. In actual environments when it is exposed to methyl iodide, simply shining a UV flashlight onto COF-1D6 allows the adsorption process to be determined by the intensity of its fluorescence without coming into contact with the adsorbent.

**Fig. 5:**
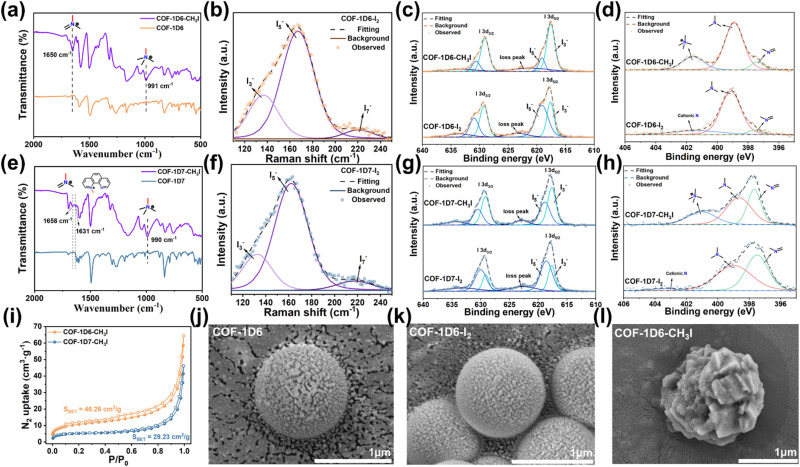
FT-IR spectra of (a) COF-1D6 and (e) COF-1D7 before and after CH_3_I adsorption. Raman spectra of (b) COF-1D6 and (f) COF-1D7. I 3d XPS and N 1s XPS spectra of (c and d) COF-1D6 and (g and h) COF-1D7 after I_2_ and CH_3_I adsorption. (i) N_2_ adsorption–desorption curves of COF-1D6 and COF-1D7 after CH_3_I adsorption. SEM images of (j) COF-1D6, (k) COF-1D6-I_2_, and (l) COF-1D6-CH_3_I.

Given that both materials exhibit excellent capture capabilities for I_2_ and CH_3_I, as well as fluorescence quenching phenomena, XRD and XPS characterizations were performed to investigate the possible adsorption mechanisms. The XRD patterns showed that the characteristic peaks of both COFs disappeared after the adsorption of I_2_ and CH_3_I (Fig. S19), and no characteristic peaks of elemental iodine appeared, indicating that the adsorbed iodine filled in the channels and interlayers of the COFs in the form of amorphous ions. Compared to raw COFs, new peaks in the Raman spectra were deconvoluted into three peaks (approximately 125, 170, and 220 cm^−1^), corresponding to I_3_^−^, I_5_^−^, and I_7_^−^ of COF-1D6-I_2_ (Fig. 5b) and COF-1D7-I_2_ (Fig. 5f), respectively. On the contrary, COFs did not exhibit new peaks in Raman spectra after CH_3_I capture (Fig. S20).

FT-IR was used to analyze the functional groups after adsorption. After adsorption of iodine vapor, the infrared peak signals of both materials were largely obscured by iodine, and no new peaks were observed (Fig. S21). However, compared with the original COFs (Fig. 5a), COF-1D6-CH_3_I exhibited signal peaks at 991 and 1650 cm^−1^ corresponding to the quaternization of sp^3^-N sites and N sites in imine bonds, respectively. The FT-IR spectrum (Fig. 5e) of COF-1D7-CH_3_I showed additional peaks at 1631 cm^−1^ corresponding to the sp^2^-N site in phenanthroline undergoing quaternization. The peak intensity of sp^3^-N^+^CH_3_I^−^ was clearly much higher than that of the other two characteristic peaks, indicating that the sp^3^-N site is the main reaction site for CH_3_I.

Consistent with the FT-IR spectra, the XPS spectra (Fig. 5d and h) of both COFs after adsorption of CH_3_I showed distinct characteristic peaks of quaternary ammonium N, while no significant N^+^ appeared after I_2_ adsorption. Therefore, based on the adsorption kinetics fitting results, the adsorption of CH_3_I by COFs depends on the quaternization reaction, which corresponds to chemical adsorption. The capture of I_2_ depends on its binding to N sites and the continuous formation of high-iodine complexes through halogen bonds, which is a physical adsorption process.

This explanation also corresponds well with the existence state of I after COFs adsorb I_2_ and CH_3_I, which mainly exist in the form of high-iodine complexes (I_3_^−^, I_5_^−^, and I_7_^−^) (Fig. 5c and g) and low-iodine ions (I^−^, I_3_^−^), respectively. The chemical reaction between COFs and CH_3_I also explains the poor cycle capacity for CH_3_I. Once saturation adsorption is reached for the first time, the N sites are occupied and cannot be physically desorbed. Therefore, during the second adsorption, there are no longer any quaternary ammonium reaction sites, resulting in a significant decrease in adsorption capacity.

Compared with I_2_ capture, the XRD characteristic peaks of the two COFs did not recover after desorption of CH_3_I, indicating that the crystal structure had changed, as shown in Fig. S22. The specific surface area of the material also significantly decreased after the first adsorption of CH_3_I, and its morphology also significantly changed, especially for COF-1D6, which has a spherical structure (Fig. 5j–l and S23). EDS and EDS mapping showed that the I_2_/CH_3_I loading capacity for both COFs is uniformly high (Fig. S24–S29).

Based on the above characterization, we used DFT calculations to further investigate the reasons for the fluorescence quenching of the two COFs and the more efficient adsorption capacity exhibited by COF-1D6. First, the fluorescence lifetimes of COF-1D6-I_2_, COF-1D7-I_2_, COF-1D6-N^+^CH_3_I^−^, and COF-1D7-N^+^CH_3_I^−^ were measured (Fig. S30 and S31). Compared with the original COFs, their fluorescence lifetime did not decrease. Combining the LUMO–HOMO energy level comparisons of the original COF fragments and I_2_ basically ruled out the photoinduced electron transfer (PET) process.

Notably, the quenching ability of I_2_ comes from the large amount of adsorption on the COF surface and the strong light-absorption ability of dark-colored iodine. Unlike this, CH_3_I is a colorless vapor, and therefore, the IFE and PET processes were essentially ruled out. We subsequently constructed quaternary ammonium sites at characteristic sites on the two COFs, and then measured the LUMO–HOMO energy levels (Fig. S32 and S33) of the molecular fragments before and after CH_3_I adsorption.

For COF-1D6, the ability to accept and donate electrons in the D and A units is similar. After the quaternization reaction occurs, the original A unit is converted into a relatively electron-rich unit, resulting in a D-A shift (Fig. S34). Therefore, excited electrons no longer accumulate in the fluorescent unit, causing fluorescence quenching. In contrast, because BPPT exhibits strong electron-withdrawing properties, COF-1D7-N^+^CH_3_I^−^ was generated and used as unit A. However, due to a decrease in electron acceptance, the excited state electrons were more concentrated in the N^+^I^−^ region, resulting in fluorescence quenching. Thus, the effect of CH_3_I affected the original ICT process, which may be the main quenching mechanism.

**Fig. 6:**
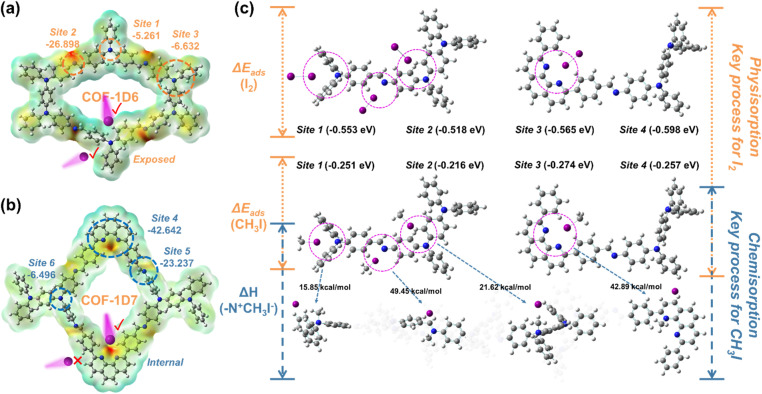
Electrostatic potential (ESP) and specific electrostatic potential energy, and exposure and internal site diagrams of (a) COF-1D6 and (b) COF-1D7. (c) Adsorption energies of I_2_ and CH_3_I at Sites 1–4 and enthalpy changes of quaternary ammonium reactions with CH_3_I.

To further illustrate the higher adsorption rate exhibited by COF-1D6, electrostatic potential (ESP) was used to reveal potential adsorption sites.^50^ As shown in Fig. 6a and b, both COFs exhibit six active nitrogen sites, namely Sites 1–3 (−5.216, −26.898, and −6.632 eV) for COF-1D6 and Sites 4–6 (−42.642, −23.237, and −6.496 eV) for COF-1D7. Given the similarities between Sites 2, 3, 5, and 6, we mainly compared the differences between Site 1 and Site 4.

As originally designed in Fig. 6a, Site 1 serves as the exposed site, and the vapor molecules of I_2_ and CH_3_I interacted with Site 1 through adsorption by either transporting in the channels of COF-1D6 or by free thermal motion in the gaps between the 1D COF bands. In contrast, N on Site 4 can only come into contact with molecules transported through the channel. The introduction of these exposed sites greatly accelerated the adsorption rate of steam molecules. In fact, the other four sites simultaneously existing in both COFs also possess the characteristics of exposed sites due to the unidirectional extension properties of 1D COFs. This is why both types of COFs exhibit high adsorption rates, with a more optimal performance from COF-1D6.

Inspired by previous reports,^37^ we also verified the advantages of exposure sites from the perspective of adsorption capacity. We assumed that when saturated adsorption has been attained, the micropores of COFs are completely filled with I_2_. Thus, the theoretical adsorption capacity was calculated from the micropore volumes of COF-1D6 (0.196 cm^3^ g^−1^) and COF-1D7 (0.180 cm^3^ g^−1^) with solid iodine density (4.93 g cm^−3^). The results were 0.966 and 0.887 g g^−1^, respectively, which is far below the actual saturation adsorption capacity of COF-1D6 (5.09 g g^−1^) and COF-1D7 (4.51 g g^−1^).

The above results strongly prove that the adsorption of iodine by the two COFs occurs not only in micropores, but more often at exposed sites that are conducive to further enrichment of iodine in the gaps between 1D COF bands. In addition to their spatial structural advantages, it has been proven that the capabilities for I_2_ and CH_3_I adsorption at all four sites are excellent. Based on DFT calculations (Fig. S35–S46), the adsorption energies (*E*_ads_) of iodine at Sites 1–4 are −0.553, −0.518, −0.565, and −0.598 eV, respectively. Additionally, the adsorption energies (*E*_ads_) of Sites 1–4 with CH_3_I were calculated as −0.251, −0.216, −0.274, and −0.257 eV, respectively.

Considering that CH_3_I adsorption is based on quaternary ammonium reactions, we additionally calculated the enthalpy changes (Δ*H*) of the quaternary ammonium reactions at Sites 1–4, which are 15.85, 49.45, 21.62, and 42.89 kcal mol^−1^, respectively. For Site 1 and Site 3 of sp^3^-N, the energy barrier of the reaction is much lower than that of sp^2^-N. This result is in accordance with the expected design, and is consistent with previous reports and infrared spectroscopy results. Therefore, the characteristics of the 1D COF exposure sites and the ultra-high sp^3^-N content indicate that COF-1D6 is the most efficient COF-type adsorbent for CH_3_I.

## Conclusions

We constructed two fluorescent N-rich 1D COFs through selection of the synthesis unit, which contains exposed sp^3^-N sites (COF-1D6) and non-exposed sp^2^-N sites (COF-1D7). The two COFs exhibited excellent capture ability for I_2_/CH_3_I due to their abundant N sites and the band topology characteristic of 1D COFs. Compared to COF-1D7 (4.51 for I_2_, 1.01 for CH_3_I), COF-1D6 exhibited higher adsorption capacities for I_2_ (5.09) and CH_3_I (1.24 g g^−1^). Using *K*_80%_ to represent the adsorption rate, COF-1D6 showed extremely high adsorption rates for I_2_ (3.77 g g^−1^ h^−1^) and CH_3_I (1.07 g g^−1^ h^−1^).

Particularly, the adsorption rate of COF-1D6 for CH_3_I is higher than that for all previously reported COF adsorbents. This outstanding property is attributed to the completely exposed sp^3^-N sites in COF-1D6, which break through the limitation of molecular transport within pores for high adsorption efficiency. The affinity and reactivity of the designed sp^3^-N with CH_3_I are also indispensable. In addition, the excellent fluorescence properties of the two COFs were judiciously applied for visual monitoring of the CH_3_I adsorption process. Based on 1D COFs, this work provides a new paradigm for improving adsorption rates by increasing the abundance of exposed sites.

## Author contributions

B. Y. revised the manuscript. K. L. designed the experiments, prepared the materials, measured the adsorption properties, collected pictures, wrote the manuscript, and completed the data simulation.

## Conflicts of interest

There are no conflicts to declare.

## Supplementary Material

SC-OLF-D5SC05993C-s001

## Data Availability

The datasets generated or analyzed during the current study are not publicly available due to the nature of the research but are available from the corresponding author on reasonable request. Additional data are made available in the Supplementary Information (SI) file. Supplementary information: the refinement details and additional figs. See DOI: https://doi.org/10.1039/d5sc05993c.
