# Supplementary material for: One-dimensional fluorescent covalent organic frameworks rich in exposed sp3-N sites for ultra-fast iodine capture and visual monitoring
Source: Chem Sci. 2025 Sep 29;16(44):20895–905. doi: 10.1039/d5sc05993c (PMC12507050; doi:10.1039/d5sc05993c)
Supplement: SC-016-D5SC05993C-s001 [file SC-016-D5SC05993C-s001.pdf]

Supplementary Information (SI) for Chemical Science.
This journal is © The Royal Society of Chemistry 2025

One-dimensional fluorescent covalent organic frameworks rich in exposed sp^3-N sites for ultra-fast iodine capture and visual monitoring

Ke Li and Bing Yan*

Shanghai Key Lab of Chemical Assessment and Sustainability, School of Chemical Science and Engineering, Tongji University, Siping Road 1239, Shanghai 200092, China.

E-mail: byan@tongji.edu.cn.

Experimental Procedure

1.Materials

N,N,N',N'-Tetrakis(4-aminophenyl)-1,4-benzenediamine (TABA) with 4,4'-diformyltriphenylamine (DTPA) and 2,9-bis[p-(formyl)phenyl]-1,10-phenanthroline (BPPT) were got from Jilin Chinese Academy of Sciences-Yanshen Technology Co., Ltd., I_2, CH_3I N, N-dimethylformamide (DMF), methanol (CH_3OH), 1,4-dioxane, ethanol (CH_3CH_2OH), tetrahydrofuran (THF), mesitylene, hydrochloric acid (HCl), NaOH were all purchased from Tansoole or Innochem. Deionized water was used throughout the experiments. All the other solvents and reagents were obtained commercially and used without further purification.

2. Synthesis of COF-1D6 and COF-1D7

DTPA and BPPT (0.2 mmol) and TABA (0.1 mmol) were dissolved in 1 mL of a 1,4-dioxane/mesitylene (1:1) mixture and then added into a Pyrex pressure vessel. Then add 0.1 mL of 6 mol/L HAc. The vessel was sealed after freeze−pump−thaw three times and then heated at 120 °C for 72 hours. The solid obtained was separated by centrifugation and washed with N,N-Dimethylformamide (DMF) and tetrahydrofuran (THF). Then, the powder was soaked in THF for 24 hours and then washed with ethanol. After this, two COFs were collected by centrifugation and dried under vacuum at 80 °C overnight.

3. Characterization instruments

Powder X-ray diffraction (PXRD) patterns were collected using a Bruker D8 Advance diffractometer with Cu K_α radiation at 40 kV and 40 mA. Fourier transform infrared spectra (FT-IR) of the powder samples were obtained on a Nicolet IS10 infrared spectrum radiometer using ATR annex. Scanning electronic microscopy (SEM) images, energy-dispersive X-ray spectra (EDS) and mapping images were obtained using ZEISS Sigma 300. Thermogravimetric analysis (TGA) was carried out on a STA8000 system analyzer under an N_2 atmosphere at a heating rate of 10 °C·min^{-1} within the temperature ranging from 25 to 800 °C. Nitrogen adsorption/desorption isotherms were measured by a Tristar 2460 analyzer at the liquid nitrogen temperature. The samples were outgassed at 120 °C for 5 h before the measurements. The Brunauer Emmett Teller (BET) method was used to calculate the surface area from the adsorption data. The pore-size-distribution curves were obtained via the non-local density functional theory (NLDFT) method. X-ray photoelectron spectroscopy (XPS) was recorded by using Thermo Scientific K-Alpha photoelectron spectrometer. The fitting curve was carried out with the XPSPEAK41 program.

4. Iodine Vapor capture

Two weighing flask (2 mL) were placed in a wide-mouth jar (250 mL) containing I_2 (2 g) or CH_3I (2mL). The adsorbent (10 mg) was placed in the first flask, while the second one was used as a reference. Then, the wide-mouth jar was sealed and kept in a heated oven at 80°C for I_2 and at room temperature for CH_3I. After a certain contact time, the wide mouth jar was taken out and cooled to room temperature. The weight of the small flask containing the resulted sample was then measured. This procedure was terminated once the weight of resulted samples named I_2@COF did not change, and the adsorption capacity (q_t, g g^{-1}) was experimentally measured by the weight increment of the resulted samples with the following equation: $Q_t=[(m_t-m_1)-(M_t-M_0)]/(m_1-m_0)$ Where q_t (g g^{-1}) represents the I_2 vapor capture uptake at time t, m_t (g) represents the weight of the vial containing adsorbents at time t, m1 (g) represents the weight of the vial containing adsorbent before sorption, m_0 (g) represents the weight of the empty vial for adsorbents, M_t (g) represents the weight of the reference vial at time t, and M_0 (g) represents the weight of the reference vial before sorption.

5. Regeneration and reusability

At room temperature, COF-1D6 and COF-1D7 (30 mg) were placed in a wide-mouth jar (250 mL) containing I_2 (5 g) or CH_3I (5mL) for 8 h, respectively. After the adsorption equilibrium was arrived, the remaining suspensions were soaked in solution of n-hexane. The desorbed material was centrifuged with deionized water and collected for regeneration. The adsorption-desorption processes were repeated 7 times.

6. Iodine capture in water and release in ethanol

Iodine capture: COFs (5 mg) was suspended in I_2 solution (50 mL, 100 ppm) from 1 to 360 min. 1 ml supernatant were withdrawn at fixed time intervals and filtered, and the removal rate of I_2 aqueous solution in the filtrate was examined by ultraviolet absorption spectroscopy compared to the absorbance of the original solution through I_t/I_0.

Iodine release: COFs@I_2 (5 mg) was suspended in I_2 solution (50 mL, 100 ppm) from 1 to 360 min. 1 ml supernatant were withdrawn at fixed time intervals and filtered, and the release process of COFs@I_2 was examined by ultraviolet absorption spectroscopy compared to the absorbance of the desorption equilibrium solution through I_t/I_e.

.

7. Theoretical calculations

The adsorption mechanisms of COF-1D6 and COF-1D7 toward I_2/CH_3I were investigated by density functional theory (DFT) calculations according to Gaussian 16 Revision.

7.1 Frontier molecular orbitals (FMO) calculations

In Frontier molecular orbitals theory, HOMO represents the highest occupied molecular orbital, while LUMO stands for the lowest unoccupied molecular orbital, respectively. The HOMO and LUMO energy levels were obtained by using Gaussian 16. Geometry optimizations and frequency calculations the smallest structural unit of COF-1D6 and COF-1D7 were performed at the B3LYP/6-311G(d) level and the optimized structure was then used to calculate single-point energy at a more precise level of B3LYP/6-311G (d, p).

The quantum mechanics calculation with density functional theory (DFT) method at B3LYP/LANL2DZ level was performed to elucidate the adsorption mechanism by Gaussian 16 software package.

Figures

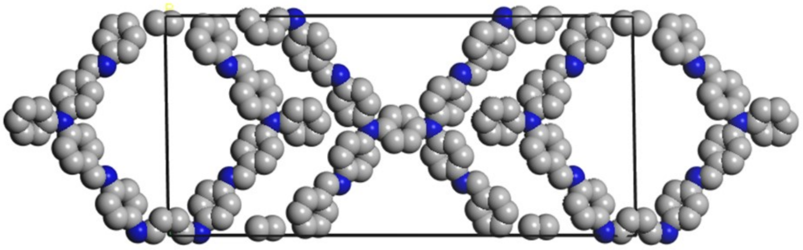

Figure S1. Structure of COF-1D6.

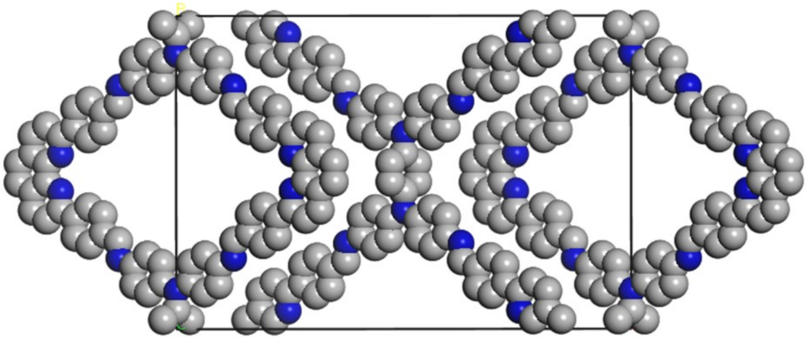

Figure S2. Structure of COF-1D7.

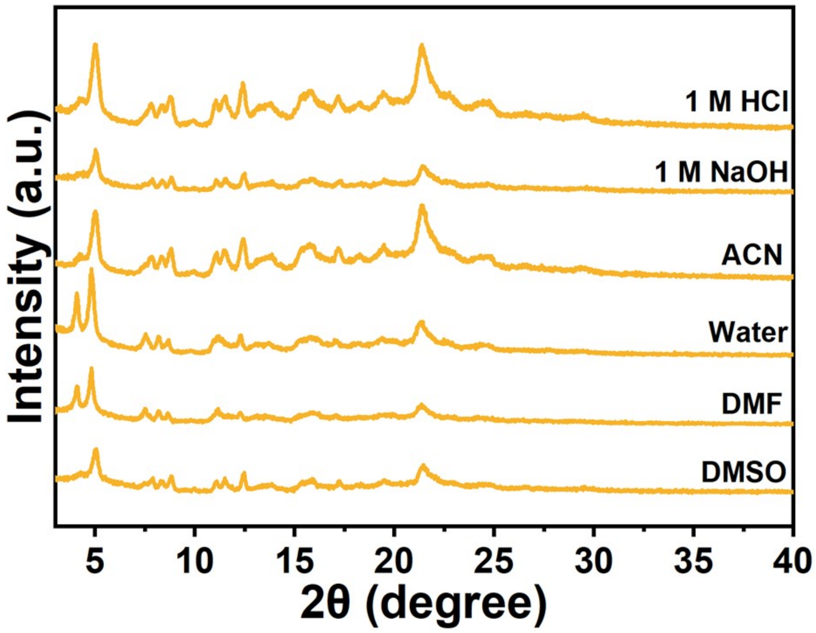

Figure S3. PXRD patterns of COF-1D6 after soaking in various solvents (water, ACN, DMSO, DMF, 1 M NaOH and 1 M HCl).

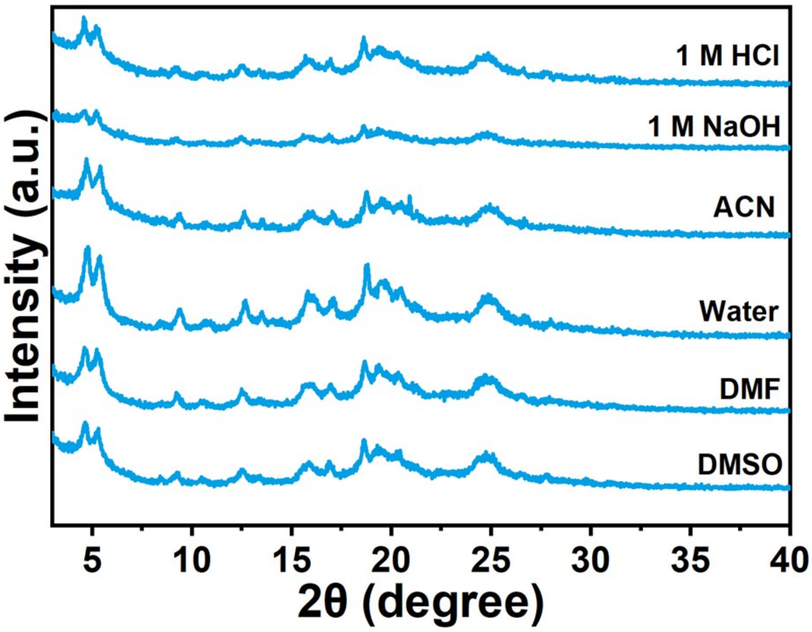

Figure S4. PXRD patterns of COF-1D7 after soaking in various solvents (water, ACN, DMSO, DMF, 1 M NaOH and 1 M HCl).

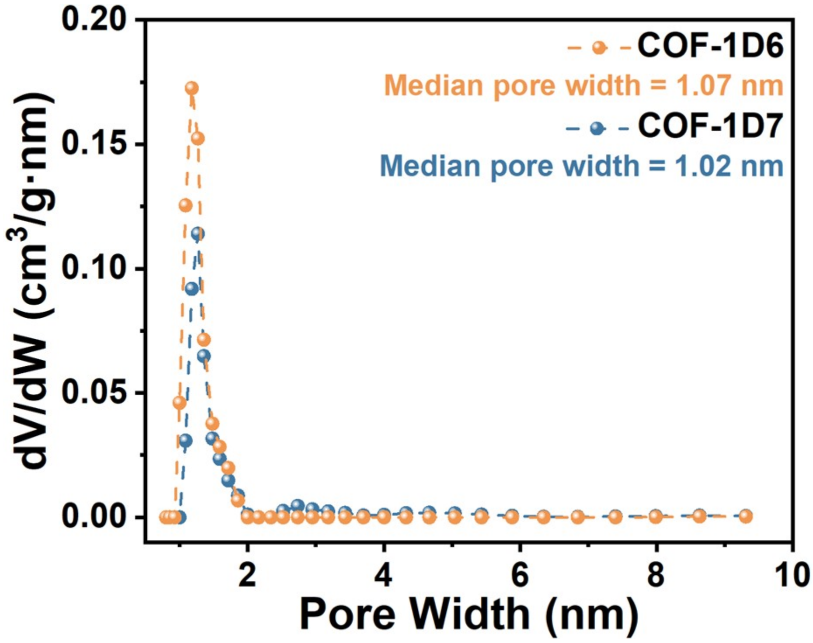

Figure S5. Pore size distribution curves of COF-1D6 and COF-1D7.

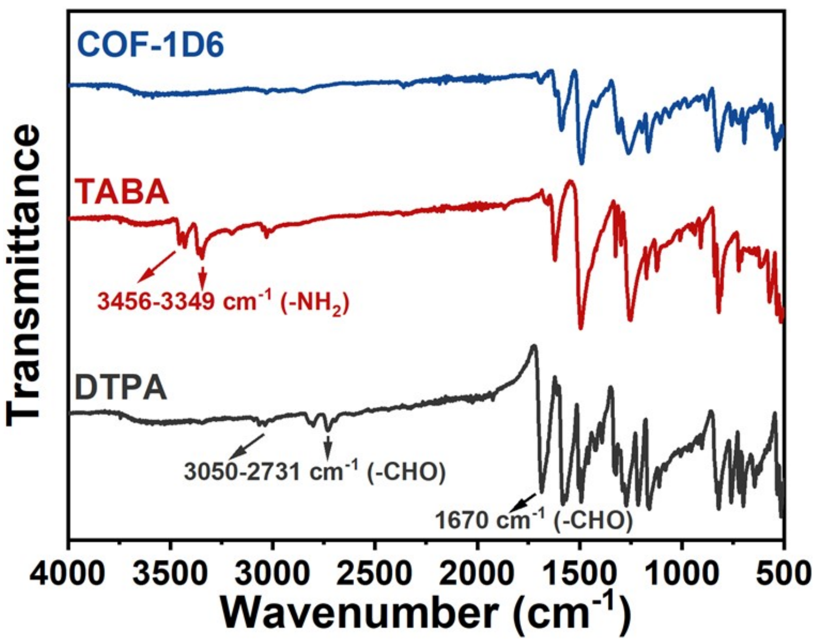

Figure S6. FT-IR spectra of COF-1D6 (blue) and its starting monomers TABA (red) and DTPA (black)

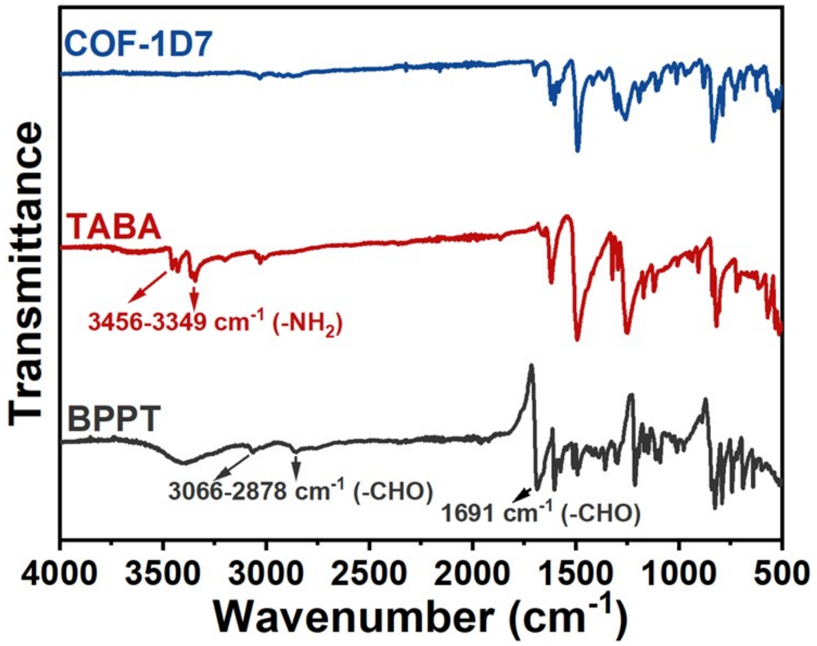

Figure S7. FT-IR spectra of COF-1D7 (blue) and its starting monomers TABA (red) and BPPT (black)

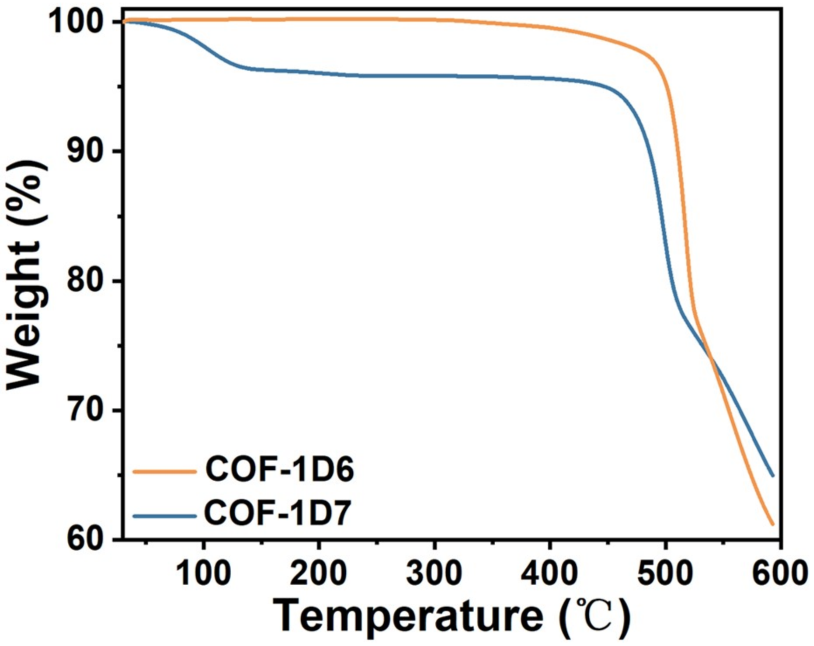

Figure S8. TGA curves of COF-1D6 and COF-1D7.

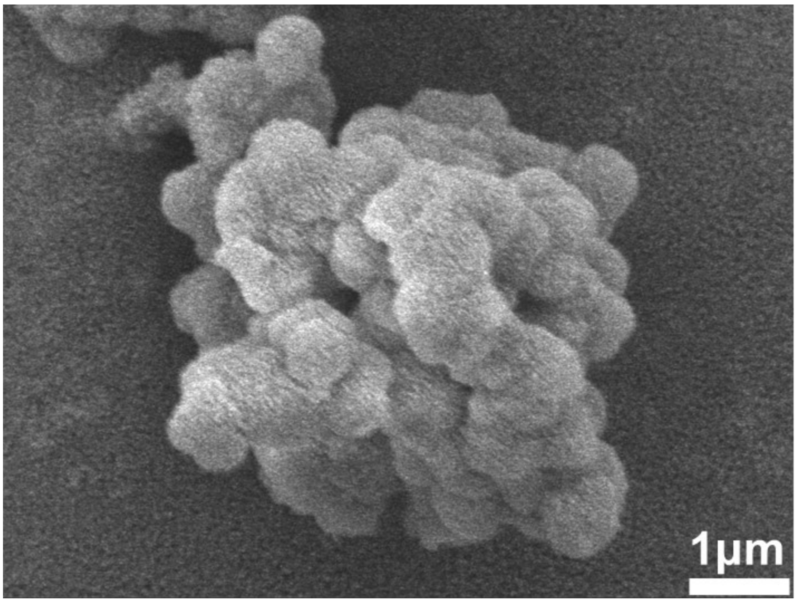

Figure S9. SEM images of COF-1D7.

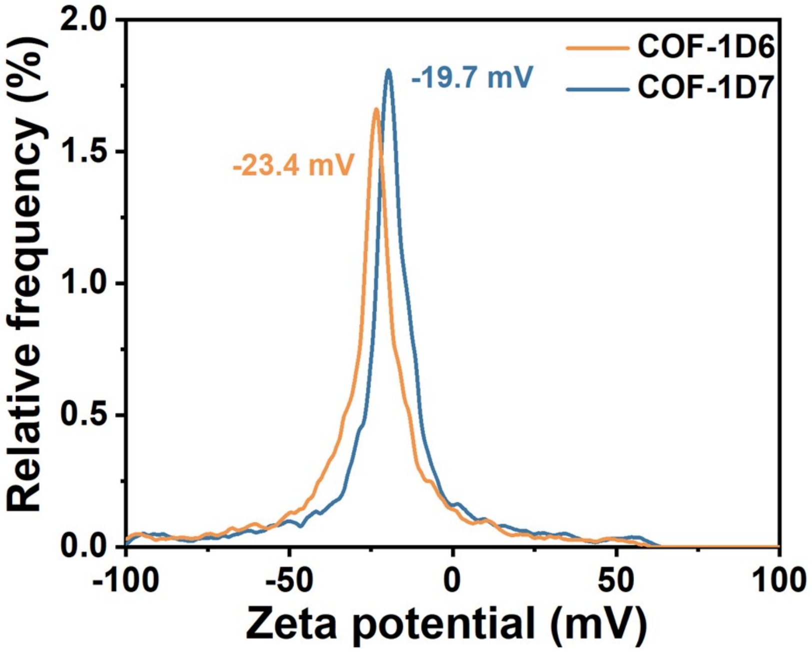

Figure S10. Zeta potentials of COF-1D6 and COF-1D7.

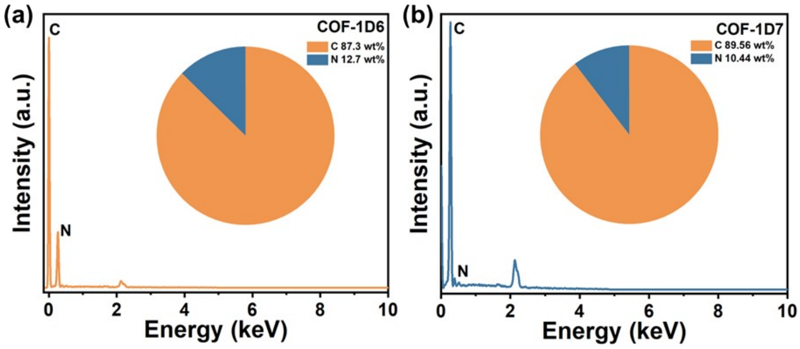

Figure S11. Elemental content of (a) COF-1D6 and (b) COF-1D7.

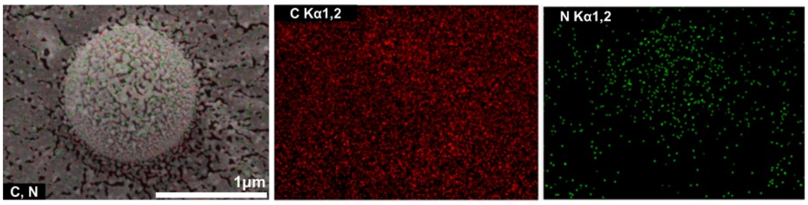

Figure S12. The EDX-mapping images of COF-1D6.

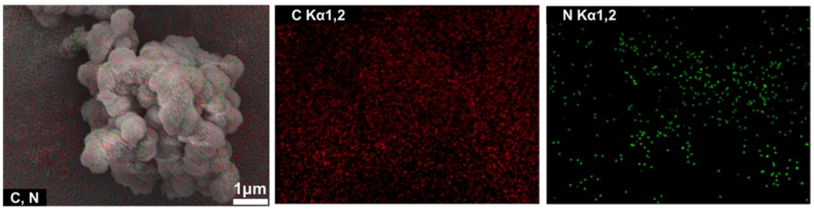

Figure S13. The EDX-mapping images of COF-1D7.

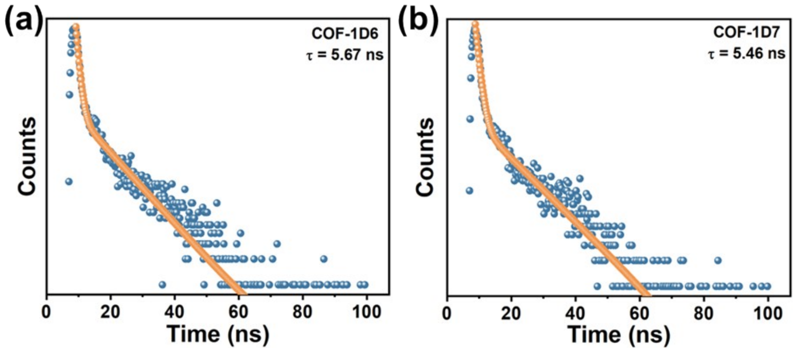

Figure S14. Fluorescence decay curves of (a) COF-1D6 and (b) COF-1D7.

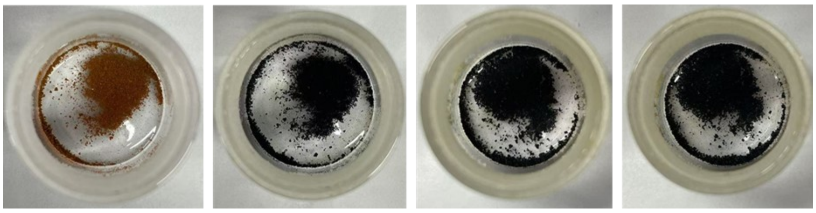

Figure S15. Photo of iodine adsorption process of COF-1D6.

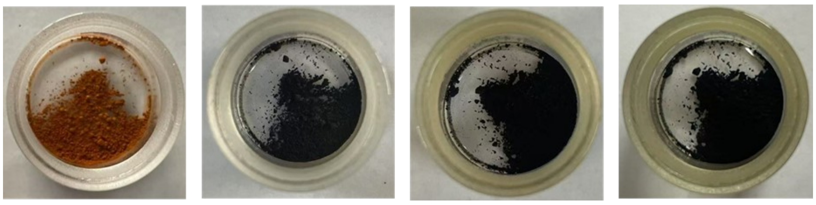

Figure S16. Photo of iodine adsorption process of COF-1D7.

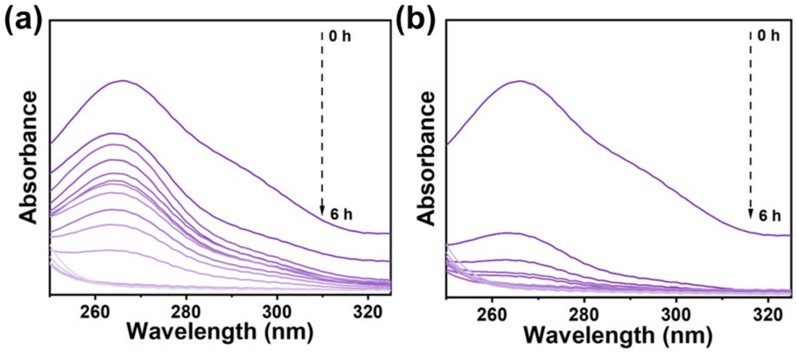

Figure S17. Removal process for I_2 in water of (a) COF-1D6 and (b) COF-1D7 through UV-Vis spectroscopy

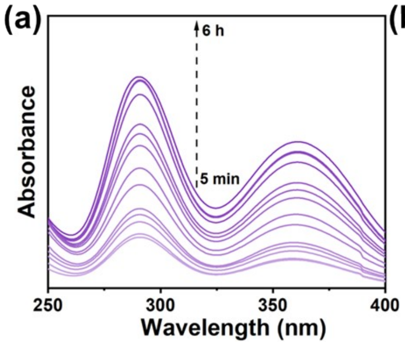

Figure S18. Desorption process of (a) COF-1D6 and (b) COF-1D7 through UV-Vis spectroscopy

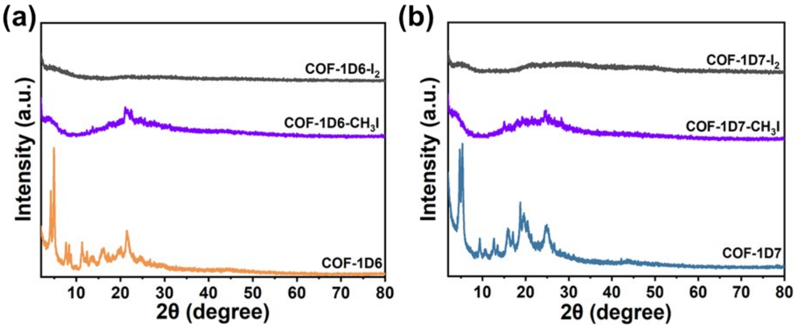

Figure S19. PXRD patterns of (a) COF-1D6 and (b) COF-1D7 after adsorption of I_2 and CH_3I.

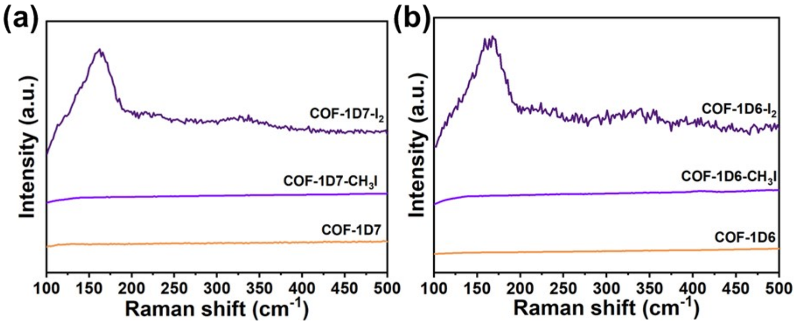

Figure S20. Raman spectra of (a) COF-1D6 and (b) COF-1D7 after adsorption of I_2 and CH_3I.

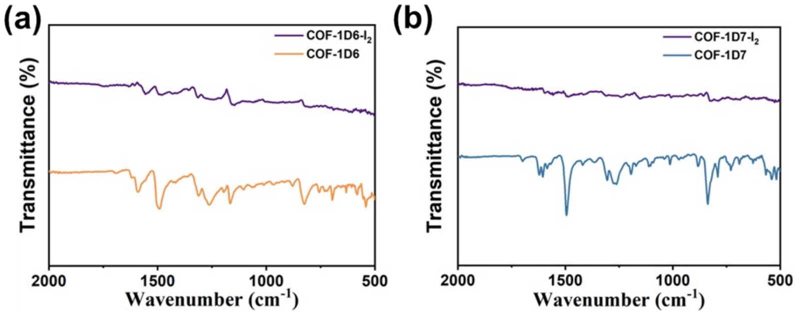

Figure S21. FT-IR spectra of (a) COF-1D6 and (b) COF-1D7 before and after I_2 adsorption.

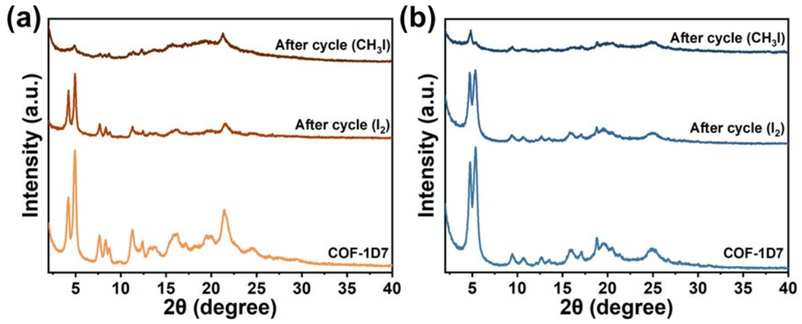

Figure S22. PXRD patterns after adsorption cycle.

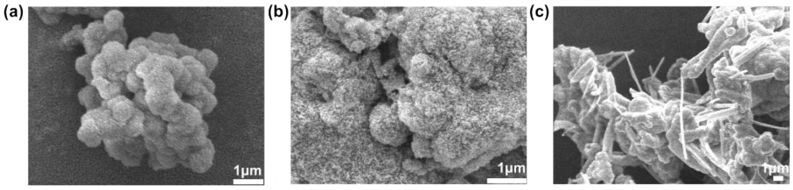

Figure S23. SEM images of (a) COF-1D7, (b) COF-1D7-I_2 and (c) COF-1D7-CH_3I.

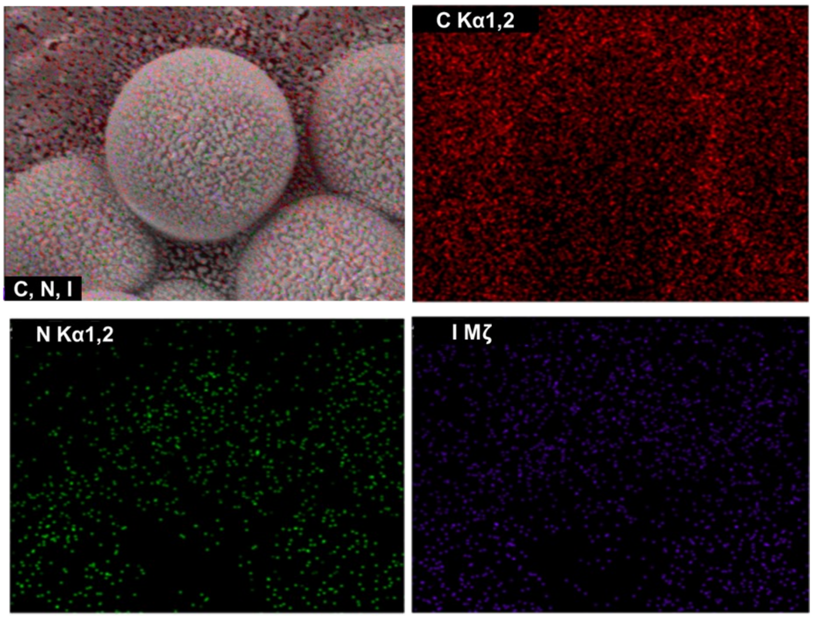

Figure S24. The EDX-mapping images of COF-1D6-I_2.

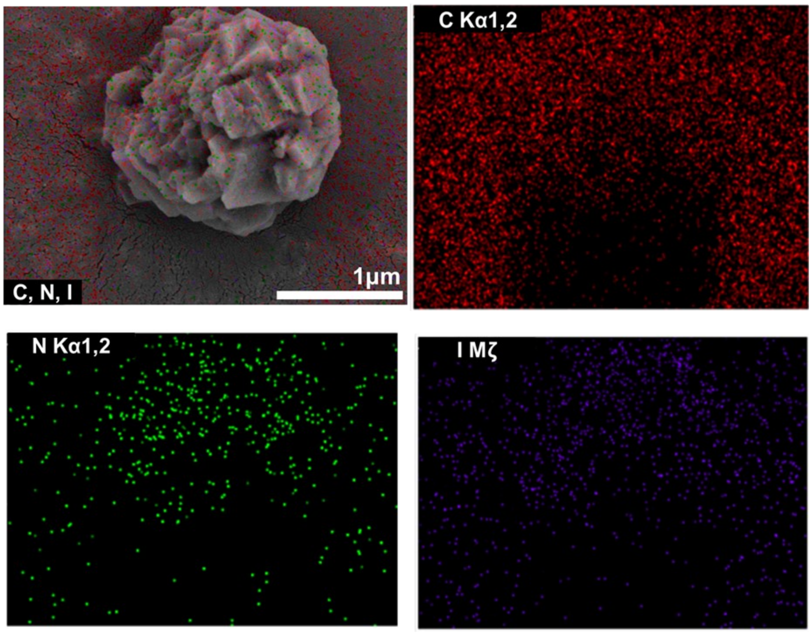

Figure S25. The EDX-mapping images of COF-1D6-CH_3I.

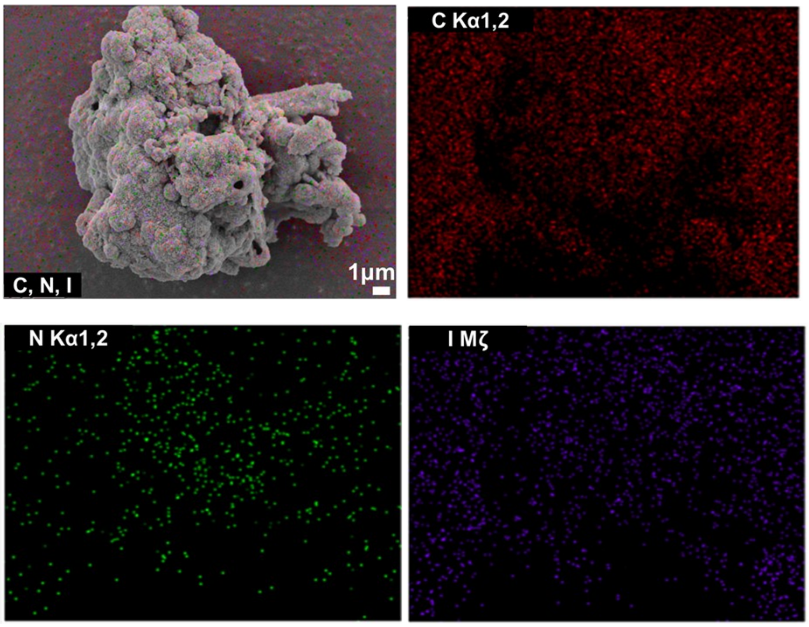

Figure S26. The EDX-mapping images of COF-1D7-I_2.

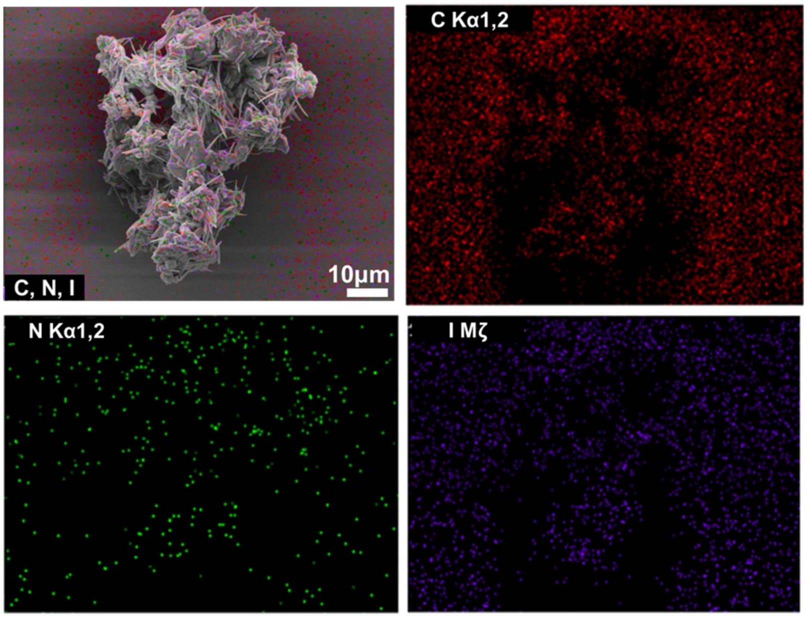

Figure S27. The EDX-mapping images of COF-1D7-CH_3I.

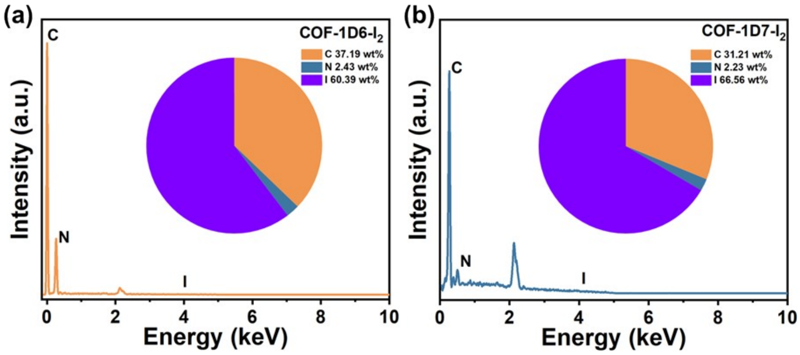

Figure S28. Elemental content of (a) COF-1D6 and (b) COF-1D7 after I_2 adsorption.

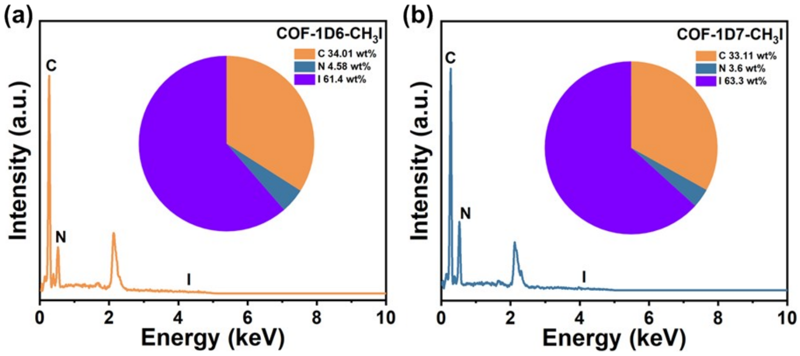

Figure S29. Elemental content of (a) COF-1D6 and (b) COF-1D7 after CH_3I adsorption.

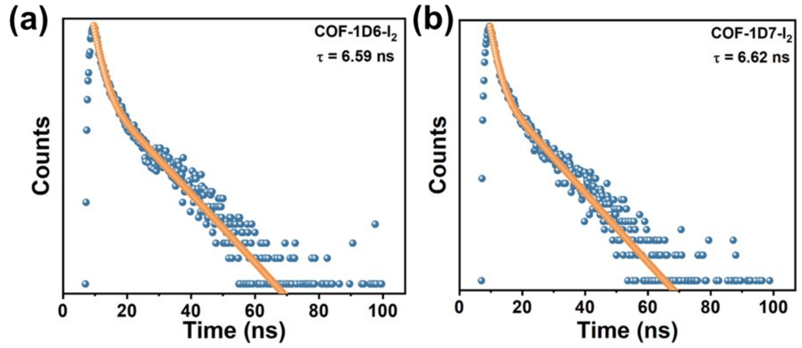

Figure S30. Fluorescence decay curves of (a) COF-1D6 and (b) COF-1D7 after I_2 adsorption

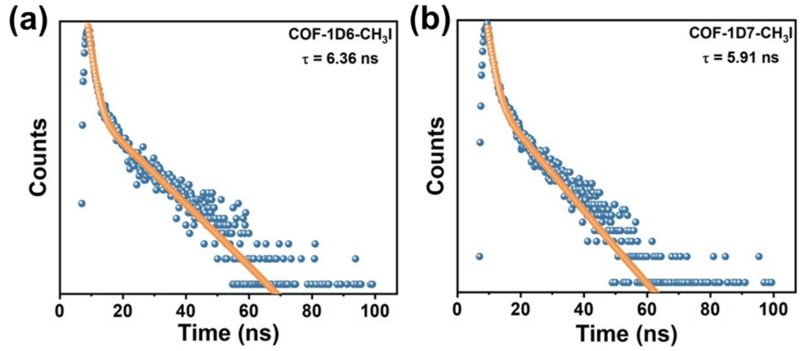

Figure 31. Fluorescence decay curves of (a) COF-1D6 and (b) COF-1D7 after CH_3I adsorption.

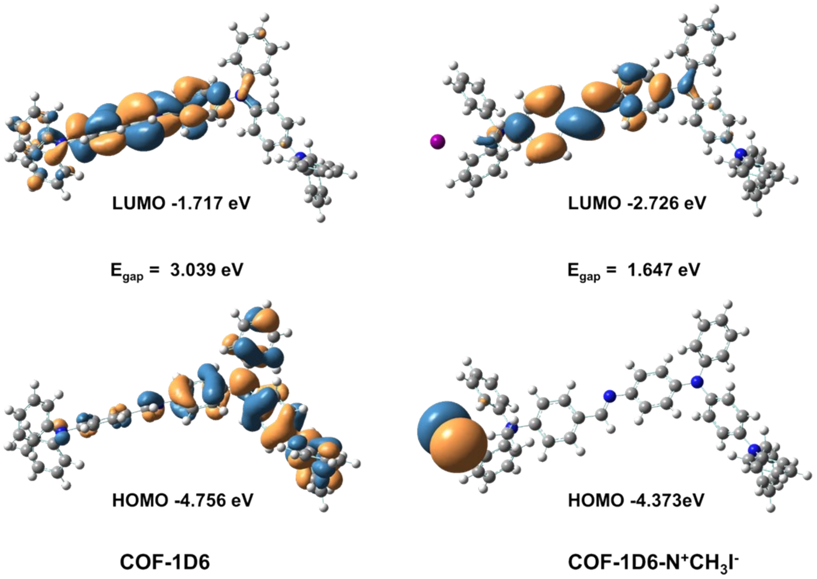

Figure S32. LUMO-HOMO energy levels of COF-1D6 and COF-1D6-$N^+CH_3I^-$

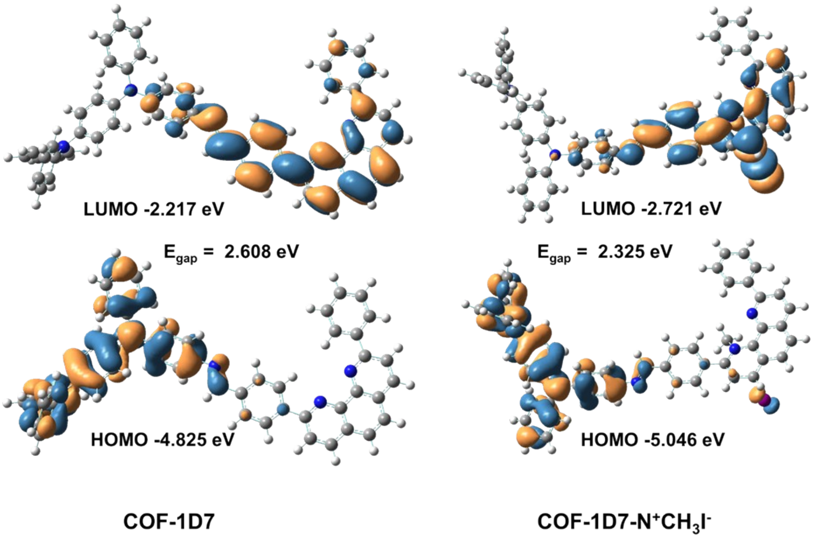

Figure S33. LUMO-HOMO energy levels of COF-1D7 and COF-1D7-$N^{+}CH_3I^{-}$

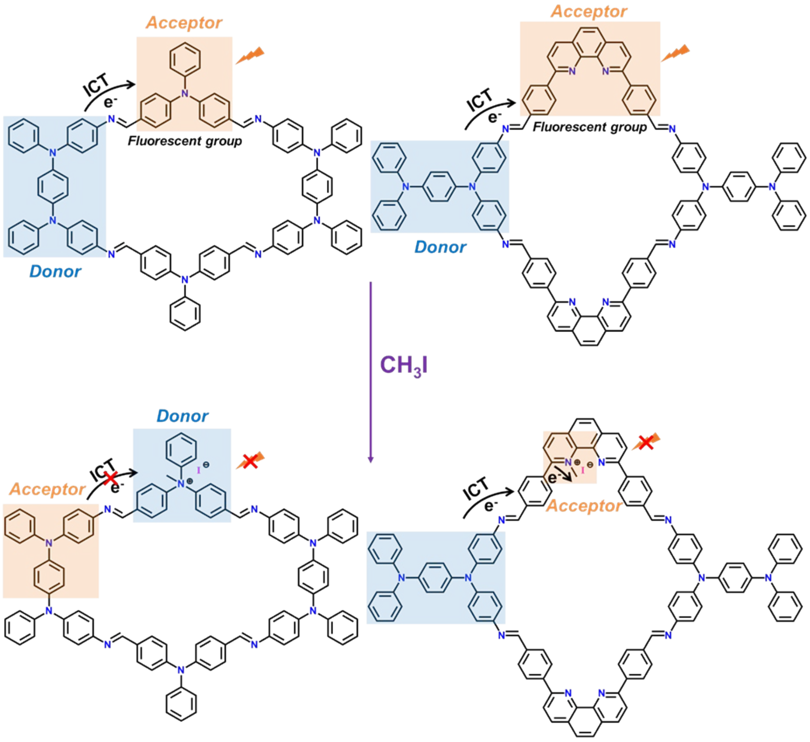

Figure S34. Fluorescence quenching processes of COF-1D6 and COF-1D7 after CH_3I adsorption.

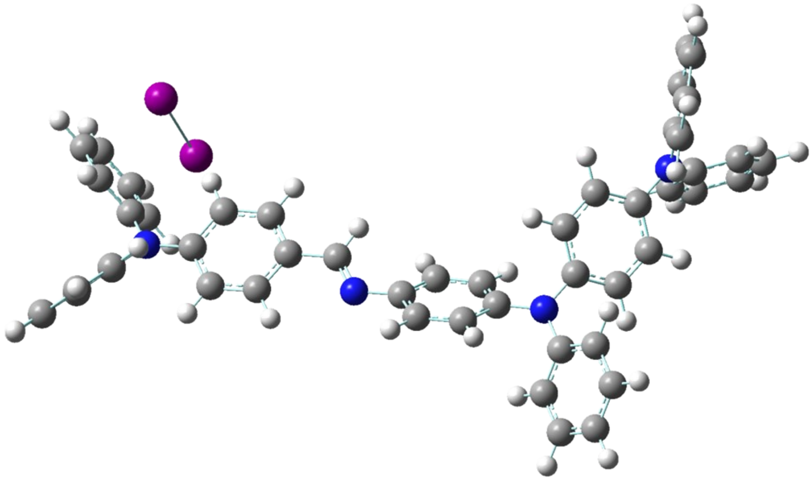

Figure S35. The raw optimized structure of adsorbing I_2 for Site 1 in COF-1D6.

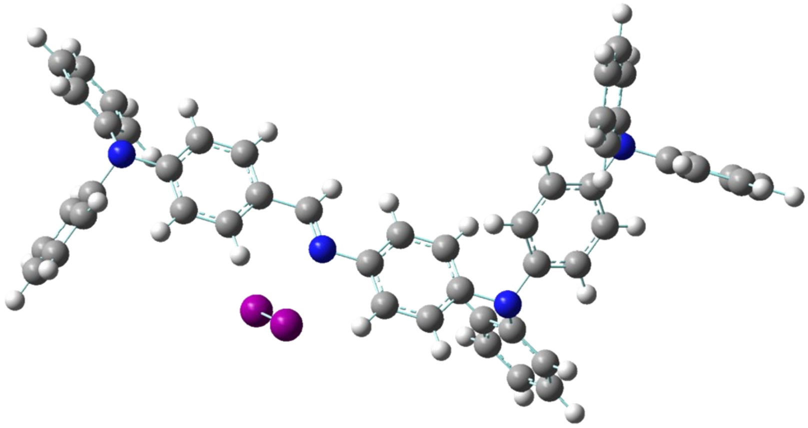

Figure S36. The raw optimized structure of adsorbing I_2 for Site 2 in COF-1D6.

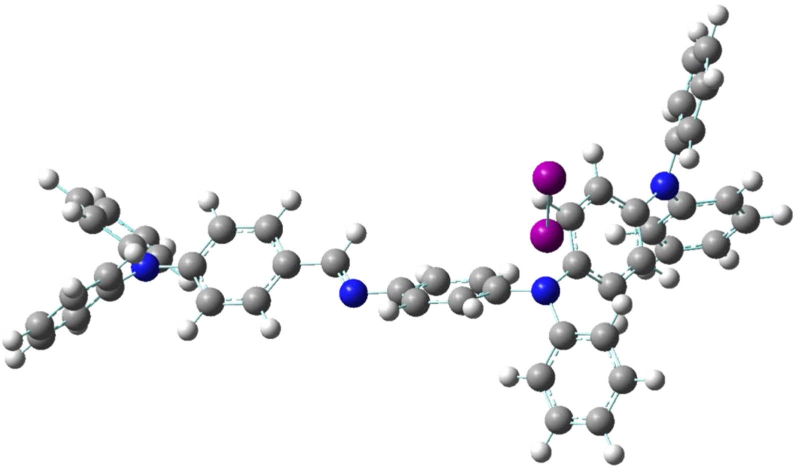

Figure S37. The raw optimized structure of adsorbing I_2 for Site 3 in COF-1D6.

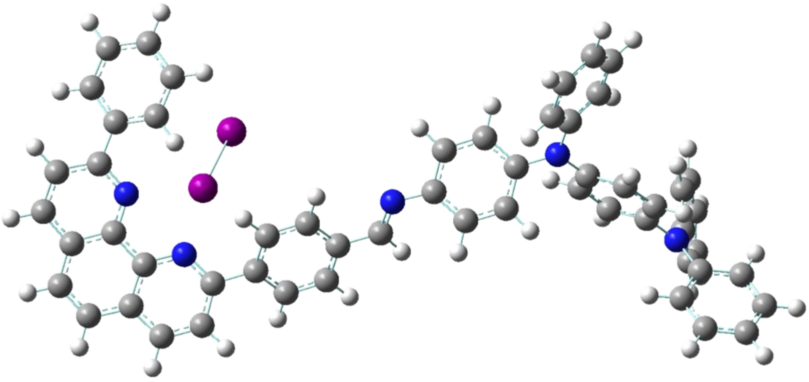

Figure S38. The raw optimized structure of adsorbing I_2 for Site 4 in COF-1D7.

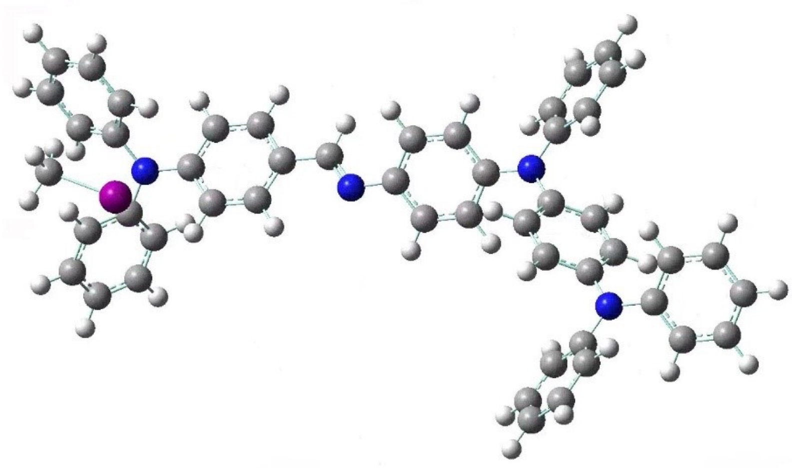

Figure S39. The raw optimized structure of adsorbing CH_3I for Site 1 in COF-1D6.

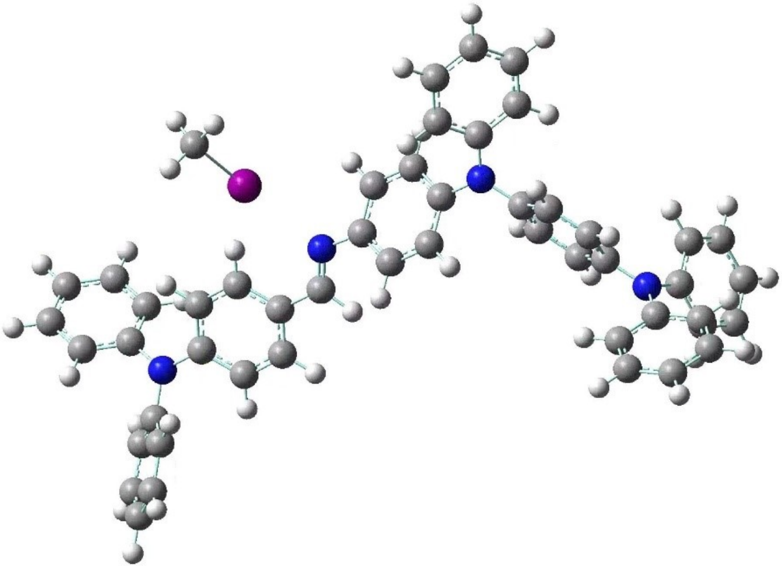

Figure S40. The raw optimized structure of adsorbing CH_3I for Site 2 in COF-1D6.

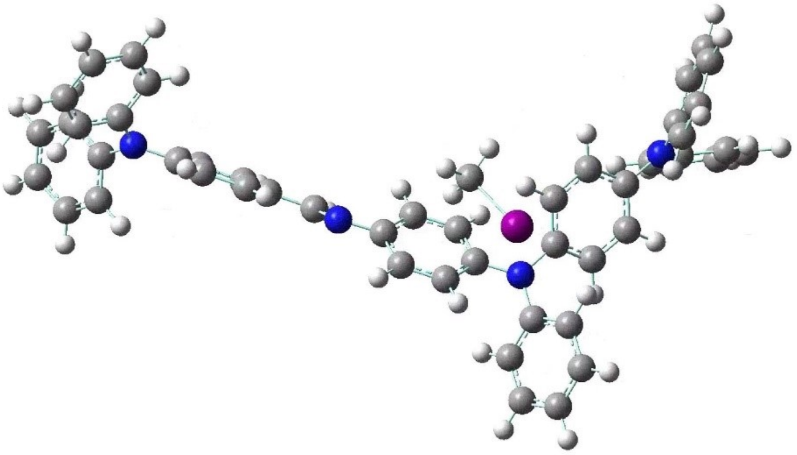

Figure S41. The raw optimized structure of adsorbing CH_3I for Site 3 in COF-1D6.

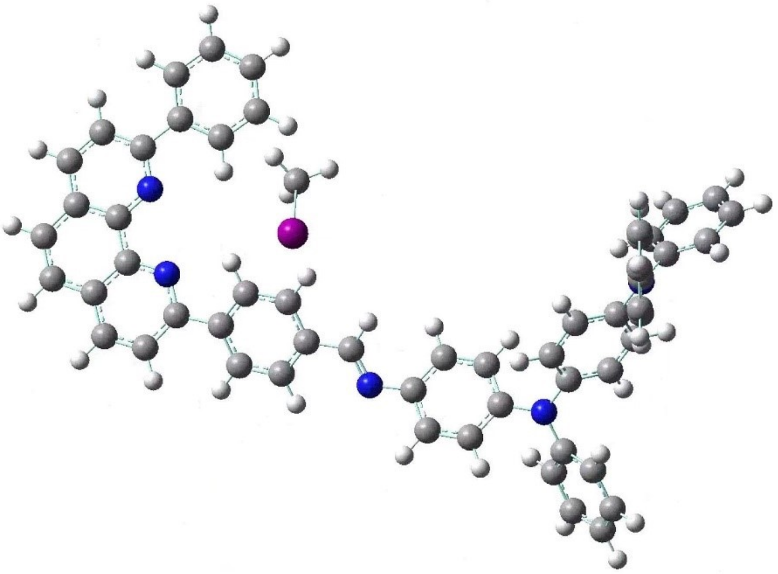

Figure S42. The raw optimized structure of adsorbing CH_3I for Site 3 in COF-1D7.

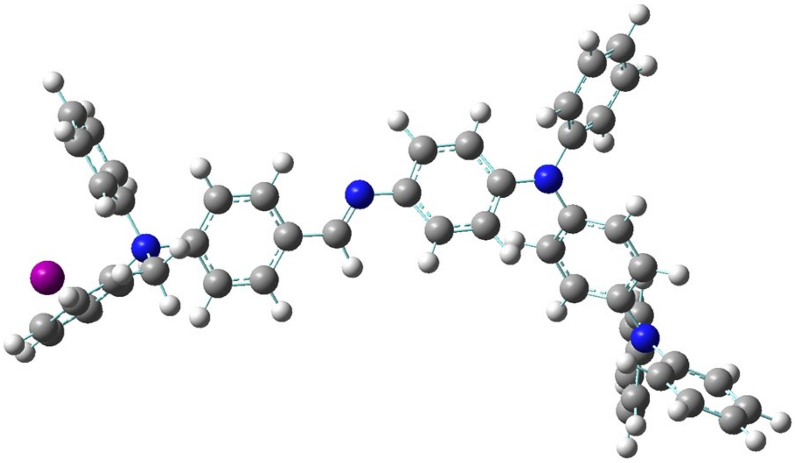

Figure S43. The raw optimized structure of COF-1D6-$N^+CH_3I^-$ for Site 1.

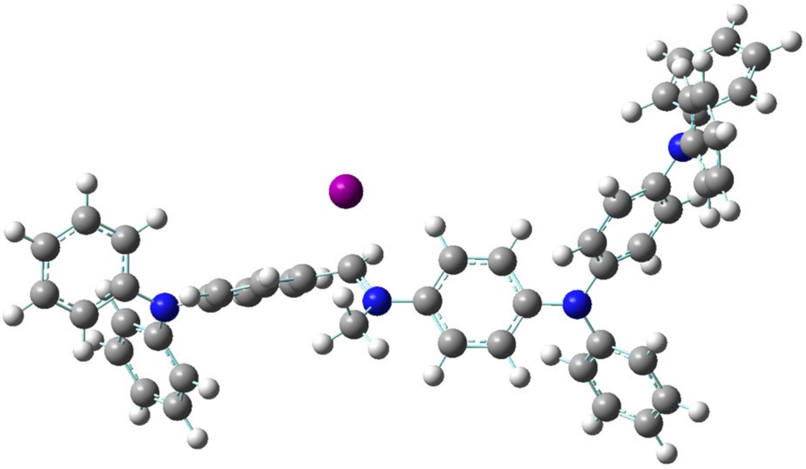

Figure S44. The raw optimized structure of COF-1D6-$N^+CH_3I^-$ for Site 2.

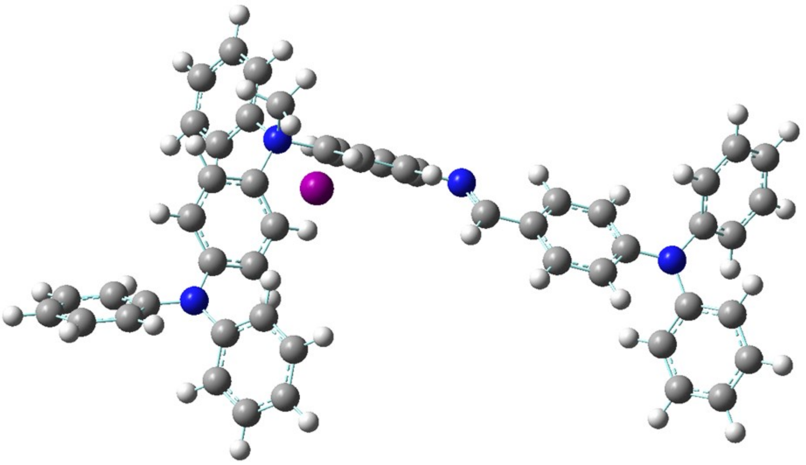

Figure S45. The raw optimized structure of COF-1D6-$N^+CH_3I^-$ for Site 3

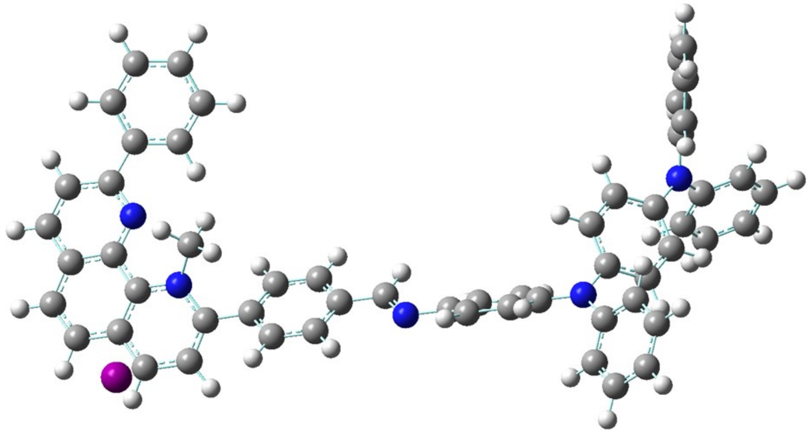

Figure S46. The raw optimized structure of COF-1D7-$N^{+}CH_3I^{-}$ for Site 4

Table

Table S1. Atomistic coordinates of COF-1D6 optimized in the Materials Studio.

Atom	x(Å)	y(Å)	z(Å)
C	0.51744	0.43171	0.28352
C	0.41197	0.29956	0.15595
C	0.38341	0.29782	0.32432
C	0.37148	0.35676	0.46736
C	0.38801	0.4164	0.44212
C	0.41666	0.41889	0.27151
C	0.42837	0.35952	0.12845
C	0.29578	0.16277	0.68812
C	0.32428	0.16593	0.51962
C	0.33671	0.10807	0.37185
C	0.32059	0.04773	0.39142
C	0.29191	0.04365	0.56151
C	0.27987	0.1022	0.71045
C	0.22848	0.92556	0.82348
C	0.19673	0.92442	0.88942
N	0.6326	0.24116	0.00333
C	0.34049	0.2301	0.50027
C	0.01312	0.96506	0.46746
C	-0.08481	0.84122	0.99383
C	-0.11507	0.83462	0.87205
C	-0.12886	0.88819	0.69719
C	-0.11274	0.9479	0.65056
C	-0.08264	0.95552	0.77981
C	-0.06875	0.90117	0.94879
C	-0.20731	0.69608	0.81581
C	-0.17663	0.70294	0.92272
C	-0.16193	0.64969	1.08998

C	-0.1778	0.58993	1.14701
C	-0.2085	0.58203	1.03499
C	-0.22306	0.63612	0.87085
C	-0.27699	0.46759	1.04187
C	-0.30873	0.46842	1.10924
N	0.13187	0.7692	0.55602
C	-0.16036	0.76568	0.85283
C	0.48111	0.5337	0.00974
C	0.58584	0.66611	0.19606
C	0.61484	0.66957	0.03836
C	0.62693	0.61271	-0.12385
C	0.61032	0.55296	-0.12281
C	0.58146	0.54852	0.04229
C	0.56936	0.60612	0.19931
C	0.70611	0.80662	-0.16785
C	0.67626	0.80093	-0.03328
C	0.66268	0.85577	0.1317
C	0.67887	0.91575	0.16176
C	0.70879	0.92238	0.02494
C	0.7222	0.86693	-0.14003
C	0.77682	0.03663	-0.05344
C	0.809	0.03636	-0.02312
N	0.36846	0.7261	0.43366
C	0.65969	0.73741	-0.06425
C	-0.01459	0.06936	0.83007
C	0.08625	0.19345	0.38143
C	0.1159	0.19713	0.52387
C	0.12864	0.14046	0.6821
C	0.1119	0.081	0.69618

C	0.08223	0.07667	0.55044
C	0.06958	0.13371	0.39374
C	0.20606	0.33545	0.74957
C	0.17665	0.32982	0.60371
C	0.16336	0.38508	0.43955
C	0.17947	0.44541	0.42137
C	0.20898	0.45197	0.56974
C	0.222	0.39603	0.73413
C	0.27436	0.56913	0.74306
C	0.30655	0.56966	0.77059
N	-0.13296	0.26414	0.85032
C	0.16023	0.26605	0.62806
C	0.4854	0.43056	0.32776
C	0.58627	0.30643	-0.11979
C	0.61591	0.30276	0.02283
C	0.62865	0.35944	0.18098
C	0.6119	0.41891	0.19483
C	0.58224	0.42322	0.04893
C	0.5696	0.36617	-0.10772
C	0.70605	0.16447	0.24973
C	0.67666	0.17007	0.10363
C	0.66337	0.11479	-0.0604
C	0.67949	0.05446	-0.07818
C	0.70899	0.04792	0.07043
C	0.722	0.10389	0.23468
C	0.77435	0.93075	0.24492
C	0.80653	0.93021	0.27298
N	0.36704	0.23581	0.34762
C	0.66023	0.23385	0.12759

C	-0.0189	0.96621	0.51216
C	0.08584	0.83376	0.69681
C	0.11483	0.83032	0.53886
C	0.1269	0.88721	0.3768
C	0.1103	0.94696	0.37823
C	0.08145	0.95138	0.54358
C	0.06936	0.89375	0.70045
C	0.20609	0.69329	0.33149
C	0.17624	0.69897	0.46627
C	0.16267	0.64411	0.6312
C	0.17886	0.58413	0.66103
C	0.20878	0.57751	0.52403
C	0.22218	0.63298	0.35907
C	0.27679	0.46325	0.44462
C	0.30897	0.46351	0.47442
N	-0.13156	0.77385	0.93596
C	0.15967	0.76249	0.43562
C	0.51314	0.53484	-0.03473
C	0.4152	0.65873	0.49131
C	0.38494	0.66533	0.36963
C	0.37115	0.61177	0.19475
C	0.38726	0.55206	0.14801
C	0.41737	0.54443	0.27717
C	0.43126	0.59877	0.44616
C	0.2927	0.80386	0.31393
C	0.32338	0.79701	0.42071
C	0.33809	0.85026	0.58792
C	0.32222	0.91002	0.64502
C	0.29152	0.91791	0.53312

C	0.27695	0.86382	0.36905
C	0.22301	0.03235	0.54055
C	0.19129	0.03153	0.60824
N	0.63188	0.73069	0.05589
C	0.33965	0.73427	0.35069
C	0.01745	0.0682	0.7856
C	-0.08802	0.20039	0.65875
C	-0.11658	0.20213	0.82711
C	-0.12851	0.14318	0.97016
C	-0.11198	0.08355	0.94491
C	-0.08333	0.08106	0.77429
C	-0.07163	0.14043	0.63124
C	-0.20423	0.33719	1.19046
C	-0.17573	0.33402	1.02207
C	-0.16329	0.39187	0.87422
C	-0.17942	0.45221	0.8936
C	-0.2081	0.4563	1.06356
C	-0.22014	0.39776	1.21259
C	-0.27155	0.57437	1.32499
C	-0.30331	0.57552	1.3906
N	0.13259	0.25873	0.50406
C	-0.15951	0.26985	1.00288
C	0.53176	0.48395	0.10246
N	0.43357	0.48139	0.24273
C	0.03176	0.01595	0.60447
N	-0.06643	0.01856	0.74542
C	0.46679	0.48154	0.19119
N	0.56507	0.48535	0.05973
C	-0.03321	0.01839	0.69368

N	0.06506	0.01455	0.5614
N	0.27523	-0.01939	0.58294
C	0.24216	-0.02035	0.64964
C	0.17818	-0.0224	0.78309
N	-0.22478	0.51933	1.08477
C	-0.25786	0.52029	1.1512
C	-0.32185	0.52234	1.28401
N	0.72558	-0.01513	0.05612
C	0.75904	-0.01599	0.08177
C	0.82382	-0.01682	0.14029
N	0.22557	0.51503	0.55502
C	0.25903	0.51587	0.58013
C	0.32382	0.51669	0.6376

Table S1. Atomistic coordinates of COF-1D7 optimized in the Materials Studio.

Atom	x(Å)	y(Å)	z(Å)
C	-0.30797	-0.11026	-0.88466
C	-0.27783	-0.14393	-0.75409
C	-0.27942	-0.20366	-0.79418
C	-0.31031	-0.22881	-0.96304
C	-0.3401	-0.19453	-1.09237
C	-0.33882	-0.13531	-1.05144
C	-0.3727	-0.21895	-1.27359
N	-0.37526	-0.2734	-1.3282
C	-0.43684	-0.26474	-1.63765
C	-0.46748	-0.29068	-1.80493
C	-0.46927	-0.35054	-1.84529
C	-0.43853	-0.38294	-1.70976
C	-0.40789	-0.3574	-1.54299
C	-0.40683	-0.29822	-1.50494

**C**	**-0.47086**	**-0.52984**	**-2.17611**
**C**	**-0.75308**	**-0.11725**	**-3.40839**
**C**	**-0.78354**	**-0.14869**	**-3.55099**
**C**	**-0.81393**	**-0.11988**	**-3.70729**
**C**	**-0.81408**	**-0.06009**	**-3.71858**
**C**	**-0.78318**	**-0.02991**	**-3.57303**
**N**	**-0.75395**	**-0.05905**	**-3.42537**
**C**	**-0.84458**	**-0.03004**	**-3.86661**
**C**	**-0.5**	**-0.56013**	**-2**
**N**	**-0.5**	**-0.62219**	**-2**

Table S3. Performance comparison between I_2 adsorbents.

Adsorbent	Adsorption rate ($K_{80\%}$ $gg^{-1}h^{-1}$)	Adsorption capacity	Ref.
**CTF-BPM-400**	**5.94**	**2.91**	**[1]**
**TAPD-PDB**	**5.34**	**7.88**	**[2]**
**TAPD-TDB**	**4.22**	**6.98**	**[2]**
**COF-1D6**	**3.77**	**5.09**	**this work**
**iCOF-TMPT**	**2.81**	**6.61**	**[3]**
**TAPT-DTDA**	**2.23**	**5.95**	**[4]**
**AC4tirmTpPaSO$_3$**	**2.1**	**4.5**	**[5]**
**TAPD-MPD**	**1.54**	**1.54**	**[2]**
**iCOF-AB-50**	**1.12**	**10.21**	**[6]**
**COF-1D7**	**0.962**	**4.51**	**this work**
**COF-TMPT**	**0.96**	**3.68**	**[3]**
**COF-N$^+$**	**0.83**	**5.11**	**[7]**
**COF-Be**	**0.614**	**3.8**	**[8]**
**COF-TAPT**	**0.48**	**8.61**	**[9]**
**OM-COF-300**	**0.3**	**3.15**	**[10]**
**TTA-DMTP-COF**	**0.17**	**2.59**	**[11]**
**NM-COF-300**	**0.13**	**1.8**	**[10]**

Table S4. Performance comparison between CH_3I adsorbents.

Adsorbent	Adsorption rate ($K_{80\%}$ $gg^{-1}h^{-1}$)	Adsorption capacity	Ref.
BIT-61	1.22	0.83	[12]
MHP-P5Q	1.12	0.8	[13]
COF-1D6	1.07	1.24	this work
COF-1D7	0.79	1.01	this work
TTA-DMTP-COF	0.21	1.6	[11]
TAPT-DTDA	0.11	2.12	[4]
COF-A	0.07	1.58	[14]
COF-B	0.06	1.37	[14]
COF-TAPT	0.04	1.51	[9]
SCU-COF-2	0.03	1.45	[15]

Reference

[1] Y. Zhao, W. Xue, Z. Jiang, D. Liu, C. Hu, H. Huang, *ACS Sustainable Chemistry & Engineering* 2023, **11**, 6741-6751.

[2] Y. Luo, Y. Qin, C. Ni, C. Liu, H. Yan, Y. Tao, W. Du, J. Zou, Chemical Engineering Journal 2024, **497**, 154941.

[3] Y. Tang, Z. He, W. Xue, H. Huang, G. Zhang, *Chemical Engineering Journal* 2023, **470**, 144211.

[4] W. Wang, T. Xu, H. Bian, L. Yin, N. Zhang, *Journal of Solid State Chemistry* 2025, **345**, 125222.

[5] J. Fu, J.-Y. Liu, G.-H. Zhang, Q.-H. Zhu, S.-L. Wang, S. Qin, L. He, G.-H. Tao, *Small* 2023, **19**, 2302570.

[6] Y. Xie, T. Pan, Q. Lei, C. Chen, X. Dong, Y. Yuan, J. Shen, Y. Cai, C. Zhou, I. Pinnau, et al. *Angewandte Chemie International Edition* 2021, **60**, 22432-22440.

[7] S. Zhang, D. Mei, K. Li, B. Yan, *Chemical Engineering Journal* 2025, **510**, 161825.

[8] K. Li, B Yan, *Inorganic Chemistry* 2025, **64**, 12722-12730.

[9] Y. Xie, T. Pan, Q. Lei, C. Chen, X. Dong, Y. Yuan, W. A. Maksoud, L. Zhao, L. Cavallo, I. Pinnau, et al. *Nature Communications* 2022, **13**, 2878.

[10] T. Liu, Y. Zhao, M. Song, X. Pang, X. Shi, J. Jia, L. Chi, G. Lu, *Journal of the American Chemical Society* 2023, **145**, 2544-2552.

[11] W.-Z. She, Q.-L. Wen, H.-C. Zhang, J.-Z. Liu, R. S. Li, J. Ling, Q. Cao, *ACS Applied Nano Materials* 2023, **6**, 18177-18187.

[12] D. Xu, Y. Ye, B. o. Li, R. Luo, J. Zhou, X. Ma, B. Wang, *Chemistry of Materials* 2025, **37**, 238-246.

[13] K. Jie, Y. Zhou, Q. Sun, B. Li, R. Zhao, D.-e. Jiang, W. Guo, H. Chen, Z. Yang, F. Huang, et al. *Nature Communications* 2020, **11**, 1086.

[14] S. Fajal, D. Majumder, W. Mandal, S. Let, G. K. Dam, M. M. Shirolkar, S. K. Ghosh, *Journal of Materials Chemistry A* 2023, **11**, 26580-26591.

[15] L. He, L. Chen, X. Dong, S. Zhang, M. Zhang, X. Dai, X. Liu, P. Lin, K. Li, C. Chen, et al. *Chem* 2021, **7**, 699-714.
